# Phase II study of atezolizumab with bevacizumab for non-squamous non-small cell lung cancer with high PD-L1 expression (@Be Study)

**DOI:** 10.1136/jitc-2021-004025

**Published:** 2022-02-01

**Authors:** Takashi Seto, Kaname Nosaki, Mototsugu Shimokawa, Ryo Toyozawa, Shunichi Sugawara, Hidetoshi Hayashi, Haruyasu Murakami, Terufumi Kato, Seiji Niho, Hideo Saka, Masahide Oki, Hiroshige Yoshioka, Isamu Okamoto, Haruko Daga, Koichi Azuma, Hiroshi Tanaka, Kazumi Nishino, Rie Tohnai, Nobuyuki Yamamoto, Kazuhiko Nakagawa

**Affiliations:** 1 Department of Thoracic Oncology, National Hospital Organization Kyushu Cancer Center, Fukuoka, Japan; 2 Department of Thoracic Oncology, National Cancer Center Hospital East, Kashiwa, Japan; 3 Department of Biostatistics, Yamaguchi University Graduate School of Medicine, Ube, Japan; 4 Department of Pulmonary Medicine, Sendai Kousei Hospital, Sendai, Japan; 5 Department of Medical Oncology, Kindai University Faculty of Medicine, Osaka-Sayama, Japan; 6 Division of Thoracic Oncology, Shizuoka Cancer Center, Shizuoka, Japan; 7 Department of Respiratory Medicine, Kanagawa Cancer Center, Yokohama, Japan; 8 Department of Respiratory Medicine, National Hospital Organization Nagoya Medical Center 4-1-1, Nagoya, Japan; 9 Department of Thoracic Oncology, Kansai Medical University Hospital, Hirakata, Japan; 10 Research Institute for Diseases of the Chest, Kyushu University Hospital, Fukuoka, Japan; 11 Department of Clinical Oncology, Osaka City General Hospital, Osaka, Japan; 12 Division of Respirology, Neurology, and Rheumatology, Department of Internal Medicine, Kurume University, Kurume, Japan; 13 Department of Internal Medicine, Niigata Cancer Center Hospital, Niigata, Japan; 14 Department of Thoracic Oncology, Osaka International Cancer Institute, Osaka, Japan; 15 Department of Thoracic Oncology, Hyogo Cancer Center, Akashi, Japan; 16 Internal Medicine III, Wakayama Medical University, Wakayama, Japan

**Keywords:** clinical trials, phase II as topic, immunotherapy, lung neoplasms

## Abstract

**Background:**

PD-L1 expression on tumor cells is a marker of PD-1/PD-L1 antibody treatment efficacy for advanced non-small cell lung cancer (NSCLC). PD-L1 antibody (atezolizumab) prolongs overall survival (OS) compared with platinum doublet as first-line treatment for NSCLC with high PD-L1 expression. Bevacizumab enhanced cytotoxic agent and epidermal growth factor receptor (EGFR) tyrosine kinase inhibitor efficacy in non-squamous (NS)-NSCLC, and PD-1/PD-L1 antibodies in preclinical models.

**Methods:**

This single-arm phase II study investigated clinical benefits of adding bevacizumab 15 mg/kg to atezolizumab 1200 mg fixed dose in a first-line setting for advanced NS-NSCLC patients with PD-L1 expression ≥50% without *EGFR*/*ALK*/*ROS1* alterations. Primary endpoint was objective response rate (ORR) assessed by central review committee. Secondary endpoints were progression-free survival (PFS), duration of response (DOR), OS, and safety.

**Results:**

Of 39 enrolled patients, 33 (84.6%) had stage IV NSCLC and 36 (92.3%) had smoking history. As of March 31, 2020, no patient had a complete response and 25 patients had a partial response (ORR=64.1%, 95% CI 47.18 to 78.80). Twelve-month PFS and OS rates were 54.9% (35.65 to 70.60) and 70.6% (50.53 to 83.74), respectively. The median DOR in 25 responders was 10.4 months (4.63–not reached). The median treatment cycle was 12 (1 to 27). Nineteen patients discontinued study treatment because of disease progression (N=17) or immune-related adverse events (AEs) (N=2) (sclerosing cholangitis or encephalopathy). There were 23 serious AEs in 12 patients, but no grade 4/5 toxicity.

**Conclusions:**

Atezolizumab with bevacizumab is a potential treatment for NS-NSCLC with high PD-L1 expression.

**Trial registration number:**

JapicCTI-184038.

## Introduction

Immune checkpoint inhibitors PD-1 antibody and PD-L1 antibody are key drugs for the treatment of driver mutation-free non-small cell lung cancer (NSCLC).^1^ PD-L1 expressed on tumor cells (TCs) and immune cells (ICs) in tumor tissues assessed with anti-PD-L1 monoclonal SP142 antibody, and on TCs assessed with 22C3 antibody, predicts the therapeutic effectiveness of PD-1 and PD-L1 antibodies.^2^

Previous studies demonstrated improved survival using PD-1 antibody pembrolizumab and PD-L1 antibody atezolizumab compared with platinum-based chemotherapy. The KEYNOTE-024 study showed improved progression-free survival (PFS) and overall survival (OS) with pembrolizumab in untreated advanced NSCLC patients with PD-L1 tumor proportion score (TPS) ≥50% assessed on TCs with 22C3 antibody.^3^ Subset analysis showed significantly extended OS with pembrolizumab compared with chemotherapy in patients with TPS ≥50%.^4^ Atezolizumab in first-line, second-line, and third-line settings showed that PD-L1 expression level-dependent survival improvement was associated with PD-L1 expression on TCs or ICs assessed with anti-PD-L1 SP142 antibody, which indicates that PD-L1 expression levels can predict atezolizumab benefit.^5^ A subset analysis demonstrated that atezolizumab improved OS compared with platinum-based chemotherapy in NSCLC patients with high PD-L1 expression (PD-L1 ≥50% (TC3) or IC PD-L1 ≥10% (IC3)).^6^

The efficacy of combination therapy with PD-1/PD-L1 antibody added to chemotherapy had clinically significant improvements in PFS and OS in patients with non-squamous (NS)-NSCLC compared with chemotherapy alone, irrespective of PD-L1 expression levels, and even better outcomes in patients with high PD-L1 expression by subset analyses.^7–11^ Therefore, PD-1/PD-L1 antibody improves survival outcomes when administered as monotherapy or combination therapy with chemotherapy, which is PD-L1 expression level-dependent. However, the efficacy and safety of PD-1/PD-L1 antibody monotherapy compared with platinum-based chemotherapy plus PD-1/PD-L1 antibody in patients with high PD-L1 expression have not been investigated. Because of a lack of evidence for combination therapy, PD-1/PD-L1 monotherapy is currently recommended as standard.^12 13^

Bevacizumab, a monoclonal antibody against vascular endothelial growth factor (VEGF), demonstrated improved OS and/or PFS with acceptable toxicity and tolerability when added to platinum-based chemotherapy or epidermal growth factor receptor (EGFR) tyrosine kinase inhibitor erlotinib in patients with NS-NSCLC.^14–17^ Non-clinical studies showed VEGF inhibition with bevacizumab improved PD-1/PD-L1 antibody therapeutic efficacy by normalizing tumor vasculature, increasing T-cell infiltration, and decreasing immunosuppressive cell activity.^18–22^ Thus, bevacizumab might enhance PD-1/PD-L1 antibody efficacy and clinical studies should investigate the clinical implications of combination therapy.

As expected, non-clinical studies and a previous prospective phase II, multicenter, randomized, open-label IMmotion150 study in patients with untreated advanced or metastatic renal cell carcinoma with PD-L1 ≥1% on ICs showed that atezolizumab and bevacizumab treatment significantly extended the median PFS (14.7 months) compared with atezolizumab monotherapy (5.5 months).^23^ In addition, the subgroup analysis in that study showed that patients with PD-L1 IC3 were associated with higher efficacy.^23^ Such remarkable extension of the median PFS with atezolizumab and bevacizumab demonstrates previous non-clinical findings are applicable to clinical studies. Therefore, we tested the efficacy of atezolizumab with bevacizumab for the treatment of NSCLC.

In Japan, the Dako 22C3 antibody is the most widely used in clinical practice as a companion diagnostic indicated as an aid to identify patients with NSCLC for treatment with pembrolizumab. Survival outcomes with atezolizumab versus docetaxel in patients receiving second-line treatment for NSCLC were recently demonstrated using tumor samples obtained in a previous phase III study and Dako 22C3 antibody.^24^ In patients with PD-L1 TPS ≥50%, assessed with 22C3 antibody, the OS HR was 0.49 (95% CI 0.29 to 0.80).^25^ The results are very similar to those reported in the KEYNOTE-010 study (pembrolizumab (10 mg/kg) vs docetaxel; 0.50 (0.36 to 0.70)).^26^ Therefore, these results suggest that the PD-L1 expression level assessed with 22C3 antibody could be a marker to predict the survival outcome of patients treated with atezolizumab. In addition, the SP142 assay has lower sensitivity for determining TPSs of TCs than other PD-L1 assays including 22C3, 28-8, and SP263.^27^ Thus, we selected the 22C3 antibody for PD-L1 immunohistochemistry staining.

In this single-arm phase II study, the efficacy and safety of atezolizumab and bevacizumab combination therapy in NS-NSCLC patients with PD-L1 TPS ≥50% were investigated to determine whether phase III studies are warranted.

## Materials and methods

### Study design and patients

This @Be study was an open-label, multicenter single-arm phase II study in patients with stage IIIB/IV NSCLC, according to the eighth edition of the TNM classification of malignant tumors,^28^ or recurrent NS-NSCLC with PD-L1 TPS ≥50% without *EGFR*, *ALK*, and *ROS1* gene alterations.

Eligible patients had histologically confirmed stage IIIB/IV or postoperative recurrent NS-NSCLC with PD-L1 TPS ≥50% assessed by immunohistochemistry with Dako 22C3 monoclonal antibody (Dako North America, Carpinteria, California, USA) at local laboratories, according to standard testing practices. Other criteria included age 20–75 years at time of informed consent, Eastern Cooperative Oncology Group performance status 0 or 1, adequate hematological, hepatic, and renal functions, and life expectancy ≥3 months at time of enrollment. No previous chemotherapy for advanced disease was allowed, but postoperative adjuvant or neoadjuvant therapy ≥6 months prior to enrollment was allowed. Previous radiotherapy was allowed, but only for non-lung lesions. Patients had to have ≥1 measurable lesions based on Response Evaluation Criteria in Solid Tumors (RECIST 1.1).

Major exclusion criteria included confirmed *EGFR* mutation and *ALK*, *ROS1* fusion positive, history/presence of hemoptysis/bloody sputum, any coagulation disorder, tumor invading or abutting major blood vessels, coexistence/history of interstitial lung disease, and previous treatment with VEGF receptor inhibitors.

### Procedures

Patients were administered atezolizumab 1200 mg fixed dose followed by bevacizumab 15 mg/kg by intravenous infusion on day 1 of a 21-day cycle (@Be regimen). Patients remained on treatment until disease progression or unacceptable toxicity or until 2 years from start of treatment. Suspension of atezolizumab or bevacizumab because of adverse events (AEs) was allowed. Patients requiring suspension of atezolizumab for ≥105 days or bevacizumab for ≥42 days from the date of the previous administration, were discontinued from study treatment. Tumor lesions were assessed radiologically at baseline, every 6 weeks to 24 months, and every 9 weeks thereafter until disease progression according to RECIST 1.1. An independent review committee comprising clinicians and radiologists reviewed all tumor images and determined tumor responses and progression status. Laboratory studies including blood and urine tests were performed at every cycle day 1 and thereafter. AEs were monitored throughout the study period, sorted by System Organ Class and Preferred Terms using MedDRA/J, and graded according to the National Cancer Institute Common Terminology Criteria for Adverse Events version 4.0.

### Outcomes

The primary endpoint was objective response rate (ORR), defined as percentage of patients achieving radiologically confirmed complete response (CR) or partial response (PR), assessed by independent review committee according to RECIST JCOG version 1.1.

Secondary endpoints were PFS, defined as time from enrollment to date of radiologically confirmed disease progression according to RECIST JCOG version 1.1 or death from any cause; duration of response (DOR), defined as time from first documented best overall response CR or PR to date of radiologically confirmed disease progression or death from any cause; OS, defined as time from enrollment to death from any cause; and safety.

### Statistical analysis

Patients with previously untreated NS-NSCLC with high PD-L1 expression were monitored for clinical responses using a one-stage binomial design. The null hypothesis was that ORR ≤40% was not clinically meaningful based on a previous study in which an ORR of 44.8% was demonstrated in NS-NSCLC patients with PD-L1 TPS ≥50% in the pembrolizumab group.^3^ The alternative hypothesis was that the proportion of patients achieving ORR was at least 62% for atezolizumab with bevacizumab, estimated by at least 22% extra increase in ORR considering an expected high ORR in treatment-naïve patients with high PD-L1 expression. Therefore, a sample size of 38 patients was planned with a one-sided type I error of 0.05 and power of 80% using an exact method based on binomial distribution.

The modified intention-to-treat (mITT) population for efficacy analysis included all patients receiving ≥1 dose of study treatment and had tumor assessment at least once after enrollment. All patients receiving ≥1 dose of study treatment were included in the safety analysis population. The 90% CI for overall response was calculated with the Clopper-Pearson method. PFS, OS, and DOR were estimated using the Kaplan-Meier method, and median values and corresponding 95% CIs were calculated using the Brookmeyer-Crowley method. The best tumor percentage change from baseline was presented as a waterfall plot by the PD-L1 TPS (50%–74% and 75%–100%). Trends of changes in the sum of longest diameters of target lesions from baseline over the treatment period were presented by RECIST response. Treatment status with atezolizumab and bevacizumab, disease progression, and death events were summarized by patient as a swimmer plot.

All statistical analyses were performed with SAS V.9.4 (SAS Institute).

This study is registered with the Japan Pharmaceutical Information Center.

## Results

### Patients

Of 40 patients enrolled at 14 institutions from August 2018 to January 2020, one was ineligible and not treated with any study drug. Thirty-nine patients received ≥1 dose of study treatment and had tumor assessment at least once after enrollment. Thirty-nine patients were included in the mITT and safety analysis populations.

The median age of 39 patients was 67 years (range: 41–75); 33 (84.6%) were male, 36 (92.3%) were current/former smokers, 33 (84.6%) were stage IVA/B, and 26 (66.7%) had PD-L1 TPS 75%–100% NS-NSCLC (table 1).

**Table 1:** Patient baseline characteristics

		Total	% or range
Sex	Male	33	84.6
Age (years)	Median	67	41–75
Body weight (kg)	Median	56.1	41.0–73.2
Smoking history	Current	7	17.9
	Former	29	74.4
	Never	3	7.7
Histological type	Adenocarcinoma	37	94.9
	Other	2	5.1
Stage	IIIB	2	5.1
	IIIC	2	5.1
	IVA	18	46.2
	IVB	15	38.5
	Recurrence	2	5.1
PD-L1 TPS	50%–74%	13	33.3
	75%–100%	26	66.7
ECOG PS	0	25	64.1
	1	14	35.9
Treatment history	Surgery	6	15.4
	Radiotherapy	8	20.5

ECOG PS, Eastern Cooperative Oncology Group performance status; PD-L1 TPS, programmed death ligand 1 tumor proportion score.

### Primary endpoint

**Figure 1:**
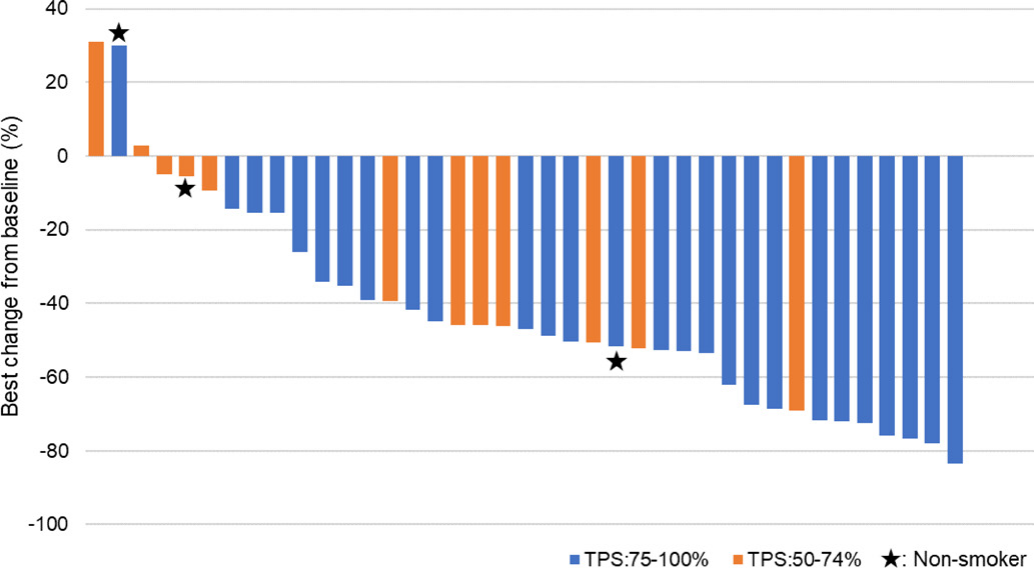
Tumor response. The best tumor percentage change from baseline in response to atezolizumab with bevacizumab by PD-L1 TPS (50%–74% and 75%–100%). Tumor responses were measured as the sum of the longest diameters of target lesions by an independent review committee. PD-L1 TPS, programmed death ligand 1 tumor proportion score.

At the data cut-off date (March 31, 2020) when tumor response evaluations were completed in 39 patients, the median follow-up was 9.5 months (IQR 5.5–12.0). Overall response was evaluable in all 39 patients, of whom none and 25 (64.1%) achieved CR and PR, respectively. The overall response was achieved in 25/39 patients (64.1%, 95% CI 47.18 to 78.80), which met the study hypothesis. Stable disease (SD) and progressive disease (PD) were found in 11 (28.2%) and 3 (7.7%) patients, respectively. No remarkable trend was observed between the best tumor percentage change from baseline and the PD-L1 TPS (figure 1). Of 39 patients, 2 SD patients and 1 PD patient were never smokers.

### Secondary endpoints

**Figure 2:**
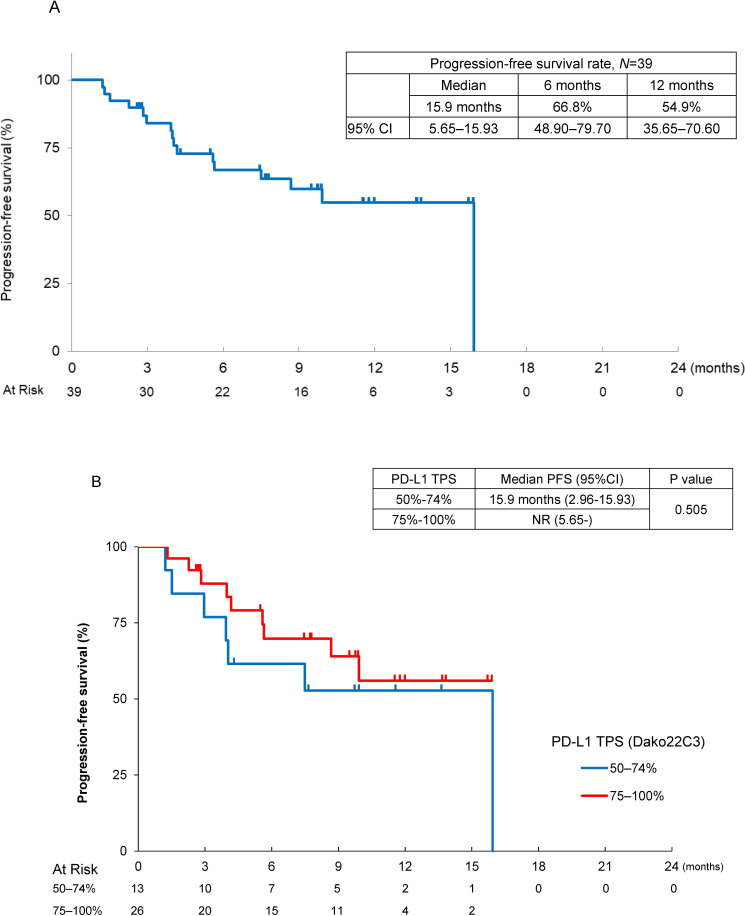
Antitumor activity and treatment status with atezolizumab with bevacizumab in non-squamous-non-small cell lung cancer patients with PD-L1 ≥50%. (A) Kaplan-Meier estimates of PFS assessed by an independent review committee. (B) Kaplan-Meier estimates of progression-free survival by PD-L1 TPS (50%–74% and 75%–100%), assessed by an independent review committee. NR, not reached; PD-L1 TPS, programmed death ligand 1 tumor proportion score; PFS, progression-free survival.

**Figure 3:**
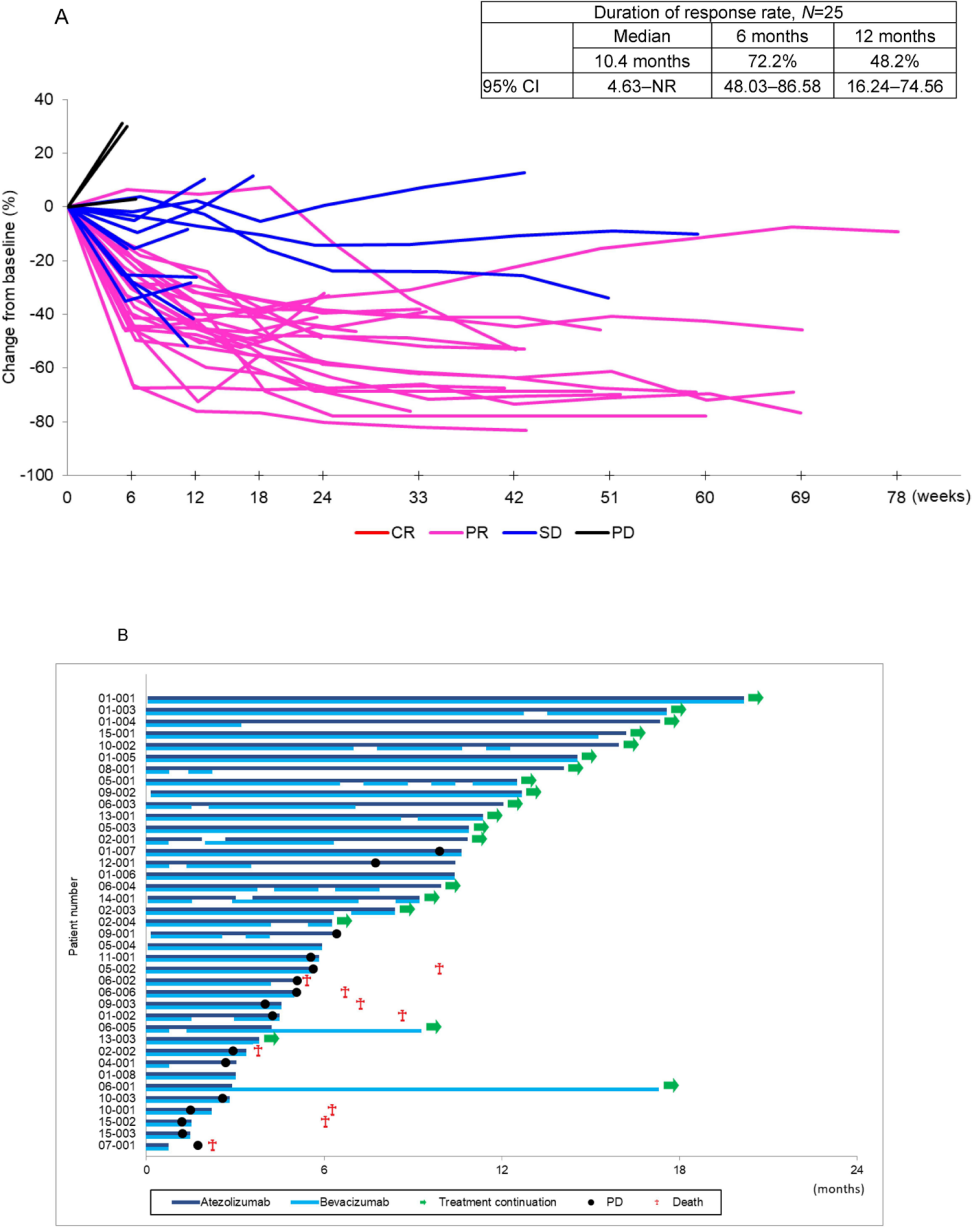
Effect of duration of treatment with atezolizumab and bevacizumab to disease progression. (A) Trend of the change in the sum of the longest diameters of target lesions from baseline over the treatment period by RECIST response. (B) Status of the study treatment, and events of disease progression and deaths. The blue bars indicating study treatment are extended to the end of treatment cycles even after a treatment discontinuation date. Patient 12-001 continued the treatment with atezolizumab even after PD at the discretion of the investigator for clinical benefit. Although the following two patients (01-008 and 05-004) discontinued the study treatment due to PD, PD symbols are not presented in the figure. Patient 01-008 with stable disease discontinued the study treatment due to worsened pain which was determined as clinical PD by the investigator. Patient 05-004 with partial response discontinued the study treatment on March 31, 2020, due to PD assessed in April 2020. Patient 07-001 discontinued the study treatment due to encephalopathy but subsequently was determined to have PD. CR, complete response; NR, not reached; NS-NSCLC, non-squamous non-small cell lung cancer; PFS, progression-free survival; PD, progressive disease; PR, partial response; RECIST, Response Evaluation Criteria in Solid Tumors; SD, stable disease.

There were 16 events in this first analysis, the median PFS was 15.9 months (95% CI 5.65 to 15.93), and 6-month and 12 month PFS rates were 66.8% (95% CI 48.90 to 79.70) and 54.9% (95% CI 35.65 to 70.60), respectively, on the basis of assessment by an independent review committee (figure 2A). By the PD-L1 TPS, the median PFS was not reached (95% CI 5.65–not reached) in patients with PD-L1 TPS 75%–100% and it was 15.9 months (95% CI 2.96 to 15.93) in patients with PD-L1 TPS 50%–74% (figure 2B). The median DOR in 25 responders was 10.4 months (95% CI 4.63–not reached), and 6-month and 12-month DOR rates assessed by independent review committee were 72.2% (95% CI 48.03 to 86.58) and 48.2% (95% CI 16.24 to 74.56), respectively (figure 3A). Durable responses were observed in some patients with PR or SD. The median cycle of treatment with atezolizumab and/or bevacizumab was 12 cycles (range 1–27) in 39 patients: 9 cycles with atezolizumab (1–27), and 8 cycles with bevacizumab (1–27). There were nine death events (29.1%), the median OS was not reached, and the 1-year survival rate was 70.6% (95% CI 50.53 to 83.74).

### Safety

At the data cut-off date, study treatment was ongoing in 20 patients (figure 3B) and had been discontinued in 19 patients: 17 for disease progression and 2 discontinued/suspended treatment for immune-related AEs (sclerosing cholangitis and encephalopathy in one patient each). Even after disease progression was determined according to RECIST 1.1, study treatment was continued in 1 (12-001, figure 3B) of 17 patients at the discretion of the investigator for clinical benefit. However, the study treatment was eventually discontinued before the data cut-off date. Of the 19 patients who discontinued the study treatment, 13 were subsequently treated with other therapies: 12 patients were treated with platinum-based chemotherapy and 1 patient was treated with palliative radiotherapy.

There were 23 serious AEs in 12 patients (30.8%), but there was no grade 4/5 toxicity. There were 33 grade 3 AEs in 15 patients (38.5%). AEs with ≥5% incidence rates were hypertension in six patients (15.4%), alanine aminotransferase increased in three (7.7%), aspartate aminotransferase increased in two (5.1%), lung infection in two (5.1%), and colitis in two (5.1%) (data not shown). Twelve atezolizumab-related serious adverse reactions were observed in nine patients (23.1%) including colitis and fever in two patients (5.1%) each (table 2). Seven bevacizumab-related serious adverse reactions were observed in six patients (all reactions in one patient each). There were no deaths during the treatment period, but nine patients died during follow-up because of disease progression.

**Table 2:** Drug-related serious adverse reactions (N=39)

	Grade 3	Grade 4–5	All grades
All, n (%)	15 (38.5)	0.0	38 (97.4)
Bronchopulmonary hemorrhage	1 (2.6)	0.0	1 (2.6)
Pericarditis	1 (2.6)	0.0	1 (2.6)
Infection	1 (2.6)	0.0	1 (2.6)
Lung infection	2 (5.1)	0.0	2 (5.1)
Hyponatremia	1 (2.6)	0.0	4 (10.3)
Encephalopathy*	1 (2.6)	0.0	1 (2.6)
Hypertension	6 (15.4)	0.0	18 (46.2)
Colitis	2 (5.1)	0.0	2 (5.1)
Diarrhea	1 (2.6)	0.0	4 (10.3)
Ileus	1 (2.6)	0.0	1 (2.6)
Anorexia	1 (2.6)	0.0	7 (17.9)
Vomiting	1 (2.6)	0.0	3 (7.7)
Cholecystitis*	1 (2.6)	0.0	1 (2.6)
Dermatitis	1 (2.6)	0.0	2 (5.1)
Proteinuria	1 (2.6)	0.0	13 (33.3)
Fever	1 (2.6)	0.0	11 (28.2)
ALT increased	3 (7.7)	0.0	8 (20.5)
AST increased	2 (5.1)	0.0	9 (23.1)
GGTP increased	1 (2.6)	0.0	3 (7.7)
ALP increased	1 (2.6)	0.0	2 (5.1)
White blood cell decreased	1 (2.6)	0.0	1 (2.6)
Neutrophil count decreased	1 (2.6)	0.0	2 (5.1)
Weight gain	1 (2.6)	0.0	2 (5.1)

*Discontinued treatment for immune-related adverse events.

ALP, alkaline phosphatase; ALT, alanine aminotransferase; AST, aspartate transaminase; GGTP, gamma-glutamyl transpeptidase.

## Discussion

This phase II study met its protocol-defined primary outcome showing 64% of patients achieved a confirmed overall response, which was higher than 38.3% ORR with atezolizumab in 107 NSCLC patients with TC3 or IC3 in the IMpower110 study.^6^ Although we should consider the NS-NSCLC patients included in this study who were expected to tolerate bevacizumab and have fewer complications, the present and previous results suggest the addition of bevacizumab to atezolizumab improved the ORR and, therefore, the @Be regimen is a promising investigational treatment for NS-NSCLC with PD-L1 TPS ≥50% compared with monotherapy. The ORR for patients with PD-L1 TPS ≥50% receiving pembrolizumab alone was 44.8% (69/154 patients) in the KEYNOTE-024 study^29^ and 39.0% (96/299 patients) in the KEYNOTE-042 study,^30^ and the addition of bevacizumab to PD-1 antibodies such as pembrolizumab or nivolumab might achieve further improvement in the ORR, based on our findings. The present ORR was comparable with 68.9% (51/74 patients) with atezolizumab and bevacizumab and carboplatin/paclitaxel in the IMpower150 study^31^ and 67.0% (59/88 patients) with carboplatin/nab-paclitaxel plus atezolizumab in the IMpower130 study^32^ in subset analyses of patients with high PD-L1 expression. Further study with an increased sample size should verify the efficacy of atezolizumab with bevacizumab compared with atezolizumab and bevacizumab and platinum-based chemotherapy.

The median PFS 15.9 months with the @Be regimen was longer than that in previous studies: 8.1 months with atezolizumab in the IMpower110 study,^6^ 12.6 months with atezolizumab and bevacizumab and carboplatin/paclitaxel in the IMpower150 study,^9^ 10.8 months with atezolizumab and pemetrexed and carboplatin or cisplatin in the IMpower132 study,^11^ and 6.4 months with atezolizumab and carboplatin and nab-paclitaxel in the IMpower130 study in patients with TC3 or IC3.^10^ Furthermore, although no statistical significance was observed because of the small sample size, our current results suggest that a better PFS may be expected in patients with PD-L1 TPS 75%–100% compared with those with 50%–74%. The 12-month PFS was 54.9% in this @Be study compared with 53.3% with atezolizumab alone in TC3 or IC3 patients in the IMpower150 study,^9^ and 46% with atezolizumab and pemetrexed and carboplatin or cisplatin in TC3 or IC3 patients in the IMpower132 study.^11^ The median PFS and 12-month PFS rate with pembrolizumab monotherapy were 10.3 months and 48% in the KEYNOTE-024 study,^29^ and 7.1 months and 37.4% in the KEYNOTE-042 subset analyses of patients with PD-L1 TPS ≥50%.^30^ Thus, the current results suggest that PFS with atezolizumab and bevacizumab is unlikely inferior to atezolizumab monotherapy or chemotherapy and atezolizumab but is likely superior to pembrolizumab monotherapy.

Our safety results demonstrated an incidence of grade 3 AEs of 38.5% but no grade 4 AE was observed. Conversely, the IMpower150 study reported incidence rates of 55.7% for grade 3–4 treatment-related AEs and 32.6% for treatment-related serious AEs including incidences of treatment-related AEs, serious treatment-related AEs, and AEs leading to withdrawal from any treatment.^9^ Although further studies are needed to verify the safety of the @Be regimen, the current results suggest that the @Be regimen is relatively safe, with few patients discontinuing treatment because of toxicity, and no new safety concerns. In this study, the median treatment cycle was 12, and 20 patients remained on treatment at the data cut-off date. Although atezolizumab or bevacizumab treatment was suspended for some patients, most continued treatment until disease progression. Thus, treatment with atezolizumab and bevacizumab appears to be tolerable and can extend the PFS in NS-NSCLC patients with PD-L1 TPS ≥50%.

At the data cut-off date, we confirmed nine deaths (23.1%) and the median OS had not been reached. Although PD-1/PD-L1 antibody treatment is expected to improve survival, the follow-up period was not long enough to confirm this. The 12-month OS of 70.6% in the current study is higher than that obtained with atezolizumab monotherapy (64.9% at 12 months)^6^ and comparable with pembrolizumab monotherapy (80.2% at 6 months).^3^ Furthermore, the @Be regimen OS is likely non-inferior to that of pembrolizumab plus platinum-based chemotherapy (73.0% at 12 months in patients with PD-L1 TPS ≥50%).^7^

Overall, current and previous studies suggest the @Be regimen improves clinical benefit without increasing the risk of new and known AEs compared with PD-1/PD-L1 antibody monotherapy for the treatment of NS-NSCLC.

Regarding the addition of PD-1/PD-L1 antibody to platinum-based chemotherapy, the efficacy of immune checkpoint inhibitors may be reduced by steroids to manage AEs and myelosuppression. Currently, there is no evidence for the negative or positive impact of platinum-based chemotherapy and concomitant drug therapies on the efficacy of immune checkpoint inhibitors. Furthermore, patients who respond to PD-1/PD-L1 antibody might not require platinum-based chemotherapy. Therefore, the non-inferiority of the @Be regimen to the PD-1/PD-L1 antibody plus platinum-based chemotherapy regimen to reduce the associated toxicity should be investigated as well as the superiority of PD-1/PD-L1 antibody with bevacizumab to monotherapy with PD-1/PD-L1 antibody alone.

Notably, 7.7% of patients had PD and the 3-month PFS was approximately 80% despite the high percentage of patients who achieved tumor responses, which indicates that 10%–20% of patients did not show sufficient clinical benefit of the @Be regimen. To identify this non-responder population, we are planning a future study using blood tumor mutational burden and gene screening.

Enrollment to this study was extended by 6 months to allow enrollment of 40 patients at 14 institutions because some high PD-L1 TPS patients were ineligible due to the risk of hemoptysis caused by tumor necrosis and cavity formation. One patient with tumor necrosis in the primary lesion determined after enrollment developed grade 1 hemoptysis after treatment initiation, which the investigator determined was related to enhanced atezolizumab efficacy by bevacizumab. This patient was treated subsequently with atezolizumab alone and achieved a PR or SD for 8 weeks until study discontinuation. Associations between presence of tumor necrosis and high PD-L1 expression were reported previously^33^; therefore, angiogenesis inhibitors should be administered to patients with high PD-L1 expression with caution.

Limitations of this study are its single-arm study design, small sample size, Japanese patients only, and short follow-up time. This study met its primary endpoint and showed secondary and safety results consistent with previous studies even under such limited conditions. A future study with a larger sample size and longer follow-up time is warranted. In addition, our ongoing biomarker study, in which tumor mutational burden status and mutations in serine/threonine kinase 11 and Kelch-like ECH-associated protein 1 are being studied, is expected to provide further information regarding the clinical benefit or lack of clinical benefit of the @Be regimen.

### Conclusion

This @Be study met the primary endpoint ORR, and the 12-month PFS, OS, and DOR results were consistent with previous studies. No new safety concerns were observed in driver mutation-free NS-NSCLC patients with PD-L1 TPS ≥50% assessed by Dako 22C3 antibody. The results of this study suggest a randomized study with a larger sample size should be initiated. The @Be regimen is a promising treatment although the superiority of atezolizumab with bevacizumab to PD-1/PD-L1 antibody monotherapy and its non-inferiority to platinum-based chemotherapy plus PD-1/PD-L1 antibody should be verified.

10.1136/jitc-2021-004025.supp1 Supplementary data

## Data Availability

Data are available upon reasonable request. Following publication deidentified individual patient data may be shared with qualifying researchers by request with a research proposal. Requests should be directed to the corresponding author.
