# Supplementary material for: Phase II study of atezolizumab with bevacizumab for non-squamous non-small cell lung cancer with high PD-L1 expression (@Be Study)
Source: J Immunother Cancer. 2022 Feb 1;10(2):e004025. doi: 10.1136/jitc-2021-004025 (PMC8808447; doi:10.1136/jitc-2021-004025)
Supplement: Supplementary data [file jitc-2021-004025supp001.pdf]

West Japan Oncology Group

[WJOG10718L]

A Phase II Study of Atezolizumab with Bevacizumab for Patients with PD-L1 High Expression Non-Small Cell Non-Squamous Cell Lung Cancer At Be Study (Investigator-initiated Clinical Study WJOG10718L)

[President of the West Japan Oncology Group (WJOG)]
Kazuhiko Nakagawa
Department of Medical Oncology, Kinki University Faculty of Medicine
[Group leader]
Nobuyuki Yamamoto
Department of Pulmonary Medicine and Oncology, Wakayama Medical University Hospital
[Coordinating investigator]
Name: Takashi Seto
Name of institution: National Hospital Organization Kyushu Cancer Center
Address: 3-1-1 Notame, Minami-ku, Fukuoka-shi, Fukuoka 813-1395
Tel: 092-541-3231 Fax: 092-551-4585
E-mail: tseto @nk-cc.go.jp

Ver.1.0 prepared on MM DD, 2018
UMIN ID: UMIN000000000

0. SYNOPSIS

0.1. SCHEMA

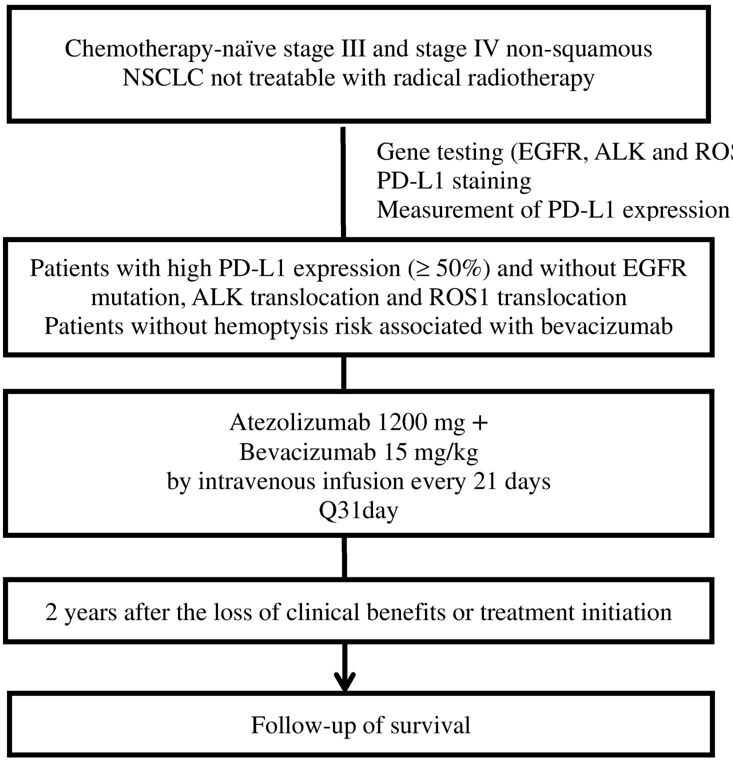

0.2. OBJECTIVES

To investigate the efficacy and safety of atezolizumab + bevacizumab therapy in treatment-naïve patients with advanced or postoperative recurrent non-squamous non-small-cell lung cancer (NSCLC) with high programmed death-ligand 1 (PD-L1) expression (tumor proportion score [TPS] ≥ 50%, Dako 22C3 antibody) and without epidermal growth factor receptor (EGFR) mutation, anaplastic lymphoma kinase (ALK) gene translocation and ROS1 gene translocation.

Primary endpoint: Objective response rate (ORR) by central assessment

Secondary endpoints: Investigator-assessed progression free survival (PFS), duration of response (DOR), overall survival (OS) and safety

0.3. SUBJECTS

1) Patients with histologically or cytologically confirmed non-squamous NSCLC
2) Patients with stage III and stage IV NSCLC not treatable with radical radiotherapy or postoperative recurrent NSCLC
3) Patients who were not previously treated with either chemotherapy or radiotherapy including treatment for other cancer types. However, patients with recurrent lung cancer at least 6 months after postoperative adjuvant chemotherapy are eligible. Patients who received radiotherapy for metastases, excluding chest irradiation, for palliative treatment are eligible. Patients with other cancer tumors, which cured only with resection (intramucosal carcinoma or having no recurrence for at least 5 years after resection), are eligible.
4) Patients with high PD-L1 expression (TPS ≥ 50%, Dako 22C3 antibody) in tumor tissue by immunohistochemistry using archived tumor tissue samples collected in the past or tissue samples taken by a biopsy at screening
5) Patients who are confirmed to be negative for EGFR mutation, ALK gene translocation and ROS1 gene translocation
6) Patients with measurable lesion as defined by the Response Evaluation Criteria in Solid Tumors (RECIST) version 1.1. However, the site of radiation is not required to be a measurable lesion.
7) Patients with Eastern Cooperative Oncology Group Performance Status (ECOG PS) 0 or 1
8) Patients without past history of interstitial pneumonia or autoimmune disease that required steroid therapy and without concurrent active interstitial pneumonia or autoimmune disease
9) Patients without hemoptysis risk factors associated with bevacizumab such as macrovascular infiltration, direct tumor penetration into the trachea or bronchus and evident tumor necrosis and cavitation

10) Patients without severe impairment of major organs and who meet criteria listed below.

The most recent data within 14 days from the date of enrollment should be used for enrollment (Data on the date 2 weeks before the date of enrollment, which is the same day of a week, are acceptable.).

Neutrophil count:	≥ 1500/mm^3
Hemoglobin:	≥ 9.0 g/dL
Platelet count:	≥ 10.0×10,000/mm^3
International normalized ratio of prothrombin time (PT-INR):	≤ 1.5
Aspartate aminotransferase (AST):	≤ 100 IU/L
Alanine aminotransferase (ALT):	≤ 100 IU/L
Total bilirubin:	≤ 1.5 mg/dL
Creatinine:	≤ 1.5 mg/dL
Percutaneous oxygen saturation (SpO_2):	≥ 90% (room air)
Urine protein:	≤ 1+

11) Patients who have personally provided written consent after receiving an adequate explanation on the contents of the clinical study prior to enrollment in the study

0.4. TREATMENT

Atezolizumab + bevacizumab therapy

Atezolizumab at a fixed dose of 1200 mg and bevacizumab 15 mg/kg will be administered as an intravenous infusion every 3 weeks (Each cycle will consist of 3 weeks, and a cycle will be repeated.) for a period during which disease progression is clinically controllable and adverse events (AEs) are manageable or for 2 years after treatment initiation.

0.5. PLANNED NUMBER OF SUBJECTS TO BE ENROLLED AND STUDY PERIOD

Planned number of subjects to be enrolled: 38 subjects
Study period: Three years from August 1, 2018 to July 31, 2021
Enrollment period: One year from August 1, 2018 to July 31, 2019
Follow-up period: Two years from the day of the enrollment of the last subject

0.6. CONTACT PERSON

Clinical Study Coordinating Secretariat
(IQVIA Services Japan K.K.?)
Mitsuru Shimomura

Tel: 080-3024-6919 Fax: 03-6859-9851
E-mail: WJOG10718L@iqvia.com

0.7. STUDY OPERATING EXPENSES

0.8. Chugai Pharmaceutical Co., Ltd. will support expenses required for operating this clinical study and supply investigational drugs (study drug and drug for the clinical study).

Table of Contents

0. SYNOPSIS 2
0.1. SCHEMA 2
0.2. OBJECTIVES 3
0.3. SUBJECTS 3
0.4. TREATMENT 4
0.5. PLANNED NUMBER OF SUBJECTS TO BE ENROLLED AND STUDY PERIOD 4
0.6. CONTACT PERSON 4
0.7. STUDY OPERATING EXPENSES 5
0.8. Chugai Pharmaceutical Co., Ltd. will support expenses required for operating this clinical study and supply investigational drugs (study drug and drug for the clinical study). 5
1. OBJECTIVES 10
2. BACKGROUND AND RATIONALES FOR THE PROTOCOL 10
2.1. Subjects 10
2.1.1. Target Disease 10
2.1.2. Rationale for the selection of the target population 11
2.2. Standard Treatment for the Subjects 12
2.3. Protocol Treatment 13
2.3.1. Protocol treatment regimen in the study 13
2.3.2. Rationale for the determination of the treatment regimen 13
2.4. Study Design 17
2.4.1. Clinical hypothesis in the study and phase setting 17
2.4.2. Rationales for the selection of endpoints 17
2.4.3. Rationale for the determination of the number of subjects to be enrolled 17
2.5. Summary of Anticipated Benefits and Disadvantages Associated with Participation in the Study 18
2.6. Planned Number of Subjects to Be Enrolled and Study Period 18
3. CRITERIA AND DEFINITIONS USED IN THE STUDY 18
3.1. Definition of Period 18
3.2. Definition of Pathological Diagnosis 19
3.3. Definition of Stage Classification 19
3.4. Definition of Response Assessment 19
3.5. Definition of Adverse Event 19
4. SELECTION OF THE SUBJECTS 19
4.1. Inclusion Criteria 19
4.2. Exclusion Criteria 21
5. SUBJECT ENROLLMENT 22
5.1. Enrollment Procedures 22
5.2. Points to Note 23
5.3. Contact Person for Enrollment 23
6. PROTOCOL TREATMENT PLAN 23
6.1. Investigational Drug and Drug for the Clinical Study 23
6.1.1. Properties of the investigational drug and the drug for the clinical study 23
6.1.2. Packaging and labels of the investigational drug and the drug for the clinical study 24

6.1.3. Storage of the investigational drug and the drug for the clinical study 24
6.1.4. Management of the investigational drug 24
6.2. Protocol Treatment 25
6.2.1. Atezolizumab + bevacizumab therapy 25
6.2.2. Administration of atezolizumab 25
6.2.3. Administration of bevacizumab 26
6.2.4. Continuation of treatment with a single drug 26
6.3. Treatment Initiation 27
6.3.1. Withdrawal before treatment 27
6.4. Completion of the Protocol Treatment 27
6.4.1. Definition of the completion of the protocol treatment 28
6.4.2. Ineffectiveness of the protocol treatment 28
6.4.3. Definitions of AEs when completing the protocol treatment 28
6.4.4. Precautions for withdrawal of consent 28
6.5. Criteria for Administration of Each Cycle (Course) and Criteria for Treatment Changes 28
6.5.1. Criteria for the start of each cycle (course) 29
6.5.2. Criteria for administration of atezolizumab in Cycle (Course) 2 and subsequent cycles (courses) 29
6.5.3. Criteria for discontinuation of atezolizumab 30
6.5.4. Criteria for resuming atezolizumab after its interruption 31
6.5.5. Criteria for administration of bevacizumab in Cycle (Course) 2 and subsequent cycles (courses) 31
6.5.6 Criteria for discontinuation of bevacizumab 32
6.6. Concomitant Therapies and Supportive Therapies 33
6.6.1 Specified concomitant therapies and supportive therapies 33
6.6.3 Prohibited concomitant therapies and supportive therapies 34
6.7. Subsequent Treatment 34
7 ENDPOINTS AND LABORATORY TESTS 34
7.1 Screening Test and Endpoints 34
7.6 Tests and Endpoints during the Protocol Treatment Period 36
7.7.1 Safety endpoints 36
7.7.2 Efficacy endpoints 37
7.8 Tests and Endpoints after the Completion of the Protocol Treatment 38
7.8.1 Safety evaluation after the end of treatment 38
7.8.2 Efficacy evaluation after the end of treatment 38
7.9 Study Schedule 39
8 DATA COLLECTION 39
8.1 Enrollment Number 39
8.2 Case Report Form (CRF) 40
8.2.1 Types of CRFs 40
8.2.2 Completing the CRF 40
8.3 Method for the Collection of CRFs 40
9 SAFETY HANDLING 41
9.1 Definition of Adverse Event (AE) 41
9.2 Definition of Serious Adverse Event (SAE) 41
9.3 Evaluation of AEs 43

9.4 Assessment of the Causal Relationship 43
9.5 Actions Taken at the Onset of AEs and Follow-up Investigation 43
9.6 AEs That Are Required to Be Reported (Reporting of SAEs) 44
9.7 Reporting to the Minister of Health, Labour and Welfare 45
9.8 Reporting to the Investigational Drug Supplier 45
9.9 Actions at the Time of Pregnancy 45
10 RESPONSE ASSESSMENT AND ENDPOINTS 46
10.1 Definitions of Endpoints 46
10.1.1 Overall survival (OS) 46
10.1.2 Progression free survival (PFS) 46
10.1.3 Objective response rate (ORR) 47
10.1.4 Duration of response (DOR) 47
11 STATISTICAL PROCEDURES AND ANALYSIS METHODS 47
11.1 Analysis Populations 47
11.2 Data Handling 48
11.2.1 Handling of protocol deviation data 48
11.2.2 Handling of missing, non-adopted and abnormal data 48
11.3 Summary of the Implementation Status of the Clinical Study 48
11.4 Baseline Characteristics 48
11.5 Efficacy Analysis 48
11.5.1 Primary efficacy endpoint 48
11.5.2 Secondary efficacy endpoints 49
11.10 Safety Analysis 49
11.11 Interim Analysis 49
12 ETHICAL MATTERS 49
12.1 Protection of the Privacy of the Subjects 49
12.2 Receiving Informed Consent 50
12.3 Matters to Be Explained to Patients Using the Written Information for Subjects 50
12.4 Preparation and Revision of the Written Information for Subjects and Informed Consent Form/ Provision of Information to the Subjects 51
12.5 Approval of the IRB 52
12.6 Review on Continuation by the IRB 52
12.7 Responsibilities and Compensation for Injuries to the Subjects 53
13 MONITORING AND AUDITS 53
13.1 Monitoring 53
13.2 Monitoring Reports 53
13.3 Audits 54
13.4 Audit Reports 54
14 QUALITY CONTROL AND ASSURANCE FOR THE STUDY 54
14.1 Data Quality Control 54
14.2 Data Quality Assurance 54
14.3 Access to Records 54
14.4 Data Handling and Retention of Records 55
14.4.1 Handling of the CRF and data 55
14.4.2 Retention of records 55
15 CHANGES, DISCONTINUATION AND TERMINATION CONCERNING THE

CONDUCT OF THE STUDY 56
15.1 Protocol Revision 56
15.2 Protocol Deviations 56
15.3 Premature Termination and Suspension of the Conduct of the Study 57
15.4 Premature Termination and Suspension at the Study Site 57
15.5 Efficacy and Safety Evaluation Committee 58
16 STUDY END AND ITS REPORTING 58
17 BURDEN OF STUDY EXPENSES 58
17.1 Study Operation Cost 58
17.2 Expenses Required for the Protocol Treatment 58
18 MATTERS CONCERNING THE CONFLICT OF INTEREST (COI) 58
19 PUBLICATION OF STUDY RESULTS AND ATTRIBUTION OF RESULTS 59
19.1 Publication of Results 59
19.2 Clinical Study Report 59
19.3 Intellectual Property Rights 59
19.4 Secondary Use of Data 60
19.5 Provision of Data 60
20 PRIOR REGISTRATION OF THE STUDY PLAN 60
21 STUDY IMPLEMENTATION STRUCTURE 60
22 OTHERS 60
23 REFERENCES 60
24 PROTOCOL REVISION HISTORY 61

1. OBJECTIVES

To investigate the efficacy and safety of atezolizumab + bevacizumab therapy in treatment-naïve patients with advanced or postoperative recurrent non-squamous non-small-cell lung cancer (NSCLC) with high programmed death-ligand 1 (PD-L1) expression (tumor proportion score [TPS] ≥ 50%, Dako 22C3 antibody) and without epidermal growth factor receptor (EGFR) mutation, anaplastic lymphoma kinase (ALK) gene translocation and ROS1 gene translocation.

Primary endpoint: Objective response rate (ORR) by central assessment

Secondary endpoints: Investigator-assessed progression free survival (PFS), duration of response (DOR), overall survival (OS) and safety

2. BACKGROUND AND RATIONALES FOR THE PROTOCOL

2.1. Subjects

2.1.1. Target Disease

In Japan, 73,838 people (52,430 men and 21,408 women) die of lung cancer each year, and the number of deaths is highest for both men and women according to the site of involvement. Lung cancer has been occupying the first position among cancer mortality since 1998. In the time-course of age-adjusted mortality rate, the number of cancer deaths has been declining, whereas the mortality rate of lung cancer remains on the same level, showing that its prognosis is still poor[1)]. Lung cancer is mainly divided into NSCLC and small cell lung cancer. NSCLC accounts for 85% of all lung cancer and is categorized according to tumor histologic types such as squamous cell cancer, adenocarcinoma and large cell cancer. Adenocarcinoma accounts for more than a half of all NSCLC, and approximately 25% is squamous cell cancer. NSCLC in the remaining patients is large cell cancer, neuroendocrine tumor, sarcomatoid cancer and poorly differentiated carcinoma.

Furthermore, NSCLC is classified into stages I to IV according to progression (Union for International Cancer Control [UICC] TNM Classification 8th Edition). For stage IA with a tumor not more than 2 cm in greatest dimension, standard treatment is radical surgery, and no postoperative chemotherapy is administered. For stage IA with a tumor 2 to 3 cm in greatest dimension, however, no evaluation of postoperative chemotherapy has been established. For stages IB, IIA, IIB and IIIA treatable with radical surgery, postoperative chemotherapy is recommended in addition to surgery. For from stage IIIA untreatable with radical surgery to stage IIIC, standard treatment is chemoradiation therapy. For stage IIIB or IIIC, to which radical radiotherapy is not indicated, and stage

IV, treatment to prolong survival and improve the quality of life (QOL) mainly with drug therapy is given.

In recent years, treatment strategies for NSCLC have been formed by dividing it into squamous cell and non-squamous cell cancers from the viewpoints of the efficacy and safety of drug therapy. Today, treatment strategies for NSCLC are built by categorizing it into driver mutation-positive NSCLC such as EGFR mutation-positive, ALK translocation-positive and ROS1 translocation-positive to which molecular-targeted drugs should be prioritized, NSCLC with high PD-L1 expression, and NSCL negative for both of these from the perspective of the indication of first-line treatment with molecular-targeted drugs and/or immune checkpoint inhibitors.

The results of a comparison between platinum-based combination therapy, a conventional standard treatment, and monotherapy with pembrolizumab, an anti-PD-1 antibody, in patients with NSCLC with high PD-L1 expression (TPS ≥ 50%, Dako 22C3 antibody) showed that pembrolizumab was significantly superior in terms of both PFS, the primary endpoint, and OS, a secondary endpoint; therefore, pembrolizumab monotherapy has been started to be recommended. Nonetheless, treatment to provide more increased clinical efficacy as maintaining patients' QOL has been awaited.

2.1.2. Rationale for the selection of the target population

2.1.2.1. Treatment-naïve patients with advanced or recurrent NSCLC with high PD-L1 expression (TPS ≥ 50%, 22C3 antibody) and without EGFR mutation, ALK gene translocation and ROS1 gene translocation

The results of a Phase III study (KEYNOTE-024) to evaluate the superiority of pembrolizumab monotherapy to platinum-based chemotherapy as a first-line treatment for treatment-naïve patients with advanced or recurrent NSCLC with high PD-L1 expression (TPS ≥ 50%, 22C3 antibody) and without EGFR mutation or ALK gene translocation were reported. Median PFS, defined as the primary endpoint, was 10.3 months in the pembrolizumab group and 6.0 months in the chemotherapy group (hazard ratio [HR] = 0.50; 95% confidence interval [CI], 0.37 to 0.68; p < 0.001). A survival rate at Month 6 was estimated to be 80.2% (95% CI, 72.9% to 85.7%) in the pembrolizumab group and 72.4% (95% CI, 64.5% to 78.9%) in the platinum-based combination therapy group. Median OS was not reached in either group but significantly longer in the pembrolizumab group than in the chemotherapy group (95% CI, 0.41 to 0.89; p = 0.005). Based on these results, pembrolizumab monotherapy was established as standard first-line treatment for patients with advanced or recurrent NSCLC with high PD-L1 expression (TPS ≥ 50%, 22C3 antibody) and without EGFR mutation and/or ALK gene translocation.

The reason why atezolizumab, an anti-PD-L1 antibody, is to be used in the present

study instead of pembrolizumab, a standard first-line treatment, is that atezolizumab alone is expected to exert an effect to prolong survival equivalent to that of pembrolizumab alone considering that the two drugs are similar: The results of a Phase III study (KEYNOTE-010) of pembrolizumab vs. docetaxel in previously treated patients with PD-L1-positive (TPS ≥ 1%, 22C3 antibody) NSCLC to compare pembrolizumab 2 mg/kg (FDA/EMA approved dose; low-dose group) with docetaxel revealed that OS, defined as the primary endpoint, was 8.5 months in the docetaxel group and 10.4 months in the pembrolizumab group (HR, 0.61; 95% CI, 0.58 to 0.88), and in a Phase III study (OAK) of atezolizumab vs. docetaxel in previously treated patients with NSCLC, OS, defined as the primary endpoint, was 10.3 months in the docetaxel group and 15.7 months in the atezolizumab group (HR, 0.74; 95% CI, 0.58 to 0.93) for the PD-L1-positive (SP142 PD-L1 ≥ 1%) population.

In addition, Dako 22C3 antibody, a companion diagnostic for pembrolizumab, which has been commonly used for PD-L1 measurements in actual clinical practice, will be employed for identifying patients with high PD-L1 expression (TPS ≥ 50%) in this study. Its reason is that the results of a study to retrospectively assess the efficacy of atezolizumab using immunohistochemical staining with Dako 22C3 antibody, utilized as a companion diagnostic for pembrolizumab, have demonstrated the effect of atezolizumab to prolong survival as compared with docetaxel in the population with PD-L1 expression ≥ 50% as determined by the 22C3 antibody.

Based on this, it is found to be appropriate to select patients who were diagnosed as having NSCLC with high PD-L1 expression (TPS ≥ 50%), as determined by the 22C3 antibody, as the study population for atezolizumab.

2.1.2.2. Non-squamous NSCLC (excluding squamous cell cancer)

The Clinical Guidelines for the Management of Lung Cancer 2017 issued by the Japan Lung Cancer Society recommend selecting treatment by dividing NSCLC into squamous cell and non-squamous cell cancers. In this study, bevacizumab, an anti-vascular endothelial growth factor (VEGF) monoclonal antibody, will be additionally administered. The INDICATION section of the package insert of bevacizumab specifies “unresectable advanced or recurrent NSCLC excluding squamous cell cancer.” This is attributable to the fact that the subjects of an overseas randomized Phase II study of bevacizumab were subsequently changed to non-squamous NSCLC due to a safety problem that all patients who died of hemoptysis had squamous NSCLC. Consequently, the target population of this study is chosen to be non-squamous NSCLC, and squamous NSCLC will be excluded.

2.2. Standard Treatment for the Subjects

For NSCLC with high PD-L1 expression (TPS ≥ 50%) as determined by Dako 22C3

antibody, pembrolizumab monotherapy (200 mg by intravenous [IV] infusion every 3 weeks) is recommended.

2.3. Protocol Treatment

2.3.1. Protocol treatment regimen in the study

Atezolizumab + bevacizumab therapy

Atezolizumab at a fixed dose of 1200 mg and bevacizumab 15 mg/kg will be administered as an IV infusion every 3 weeks (Each cycle will consist of 3 weeks, and a cycle will be repeated.) for a period during which disease progression is clinically controllable and AEs are manageable or for 2 years after treatment initiation (either on an outpatient or inpatient basis; Even if treatment with one of the two drugs is discontinued due to AEs, treatment with the other one alone may be administered.).

2.3.2. Rationale for the determination of the treatment regimen

No clinical study to investigate the usefulness of atezolizumab + bevacizumab therapy in treatment-naïve patients with advanced or recurrent non-squamous NSCLC has been reported in either Japan or foreign countries. Rationales for evaluating atezolizumab + bevacizumab therapy in the target population of this study include as follows: Based on the results of a Phase III study (OAK), the efficacy and safety of atezolizumab monotherapy as a second-line treatment for patients with advanced or recurrent NSCLC were shown; its therapeutic results similar to those of anti-PD-1 antibodies (nivolumab and pembrolizumab), which have been already approved in Japan, were reported, and thus atezolizumab monotherapy has been established as a standard treatment in Japan; and bevacizumab has been commonly employed as a standard first-line treatment for non-squamous NSCLC, and the enhancement of its clinical efficacy as a results of bevacizumab plus platinum-based combination therapy or in combination with molecular-targeted drugs have been notified.

For a clinical study of atezolizumab, a Phase III study (OAK) to investigate the superiority of atezolizumab to docetaxel in previously treated patients with advanced or recurrent NSCLC has been reported. Median OS, defined as the primary endpoint, was 13.8 months in the atezolizumab group and 9.6 months in the docetaxel group, showing a significant prolongation effect (HR = 0.73; 95% CI, 0.62 to 0.87; p = 0.0003).

This OAK Study evaluated the concordance between the 22C3 antibody and SP142 antibody in patients with remaining tissue samples that could be stained with Dako 22C3 antibody, and the majority (77%) of patients who were PD-L1-negative as determined by the SP142 antibody was also PD-L1-negative as assessed by the 22C3 antibody. The report suggested that therapeutic response to atezolizumab could be

interpreted from PD-L1 expression measured using the 22C3 antibody that the HR for survival in the atezolizumab group vs. the docetaxel group was 0.37 (95% CI, 0.20 to 0.66) in patients with high PD-L1 expression (PD-L1 tumor cells [TC] ≥ 50% or tumor-infiltrating immune cells [IC] ≥ 10%) as determined by immunohistochemical staining using Ventana SP142 antibody, and the HR in the atezolizumab group vs. the docetaxel group was 0.49 (95% CI, 0.29 to 0.80) in the subgroup with high PD-L1 expression (TPS ≥ 50%) as determined by immunohistochemical staining using Dako 22C3 antibody.

In a Phase III study (KEYNOTE-010) to evaluate the superiority of pembrolizumab to docetaxel in previously treated patients with NSCLC with TPS ≥ 1% as determined by immunohistochemical staining using the 22C3 antibody, a subset analysis in the subgroup with TPS ≥ 50% revealed that median survival was 17.3 months for pembrolizumab 10 mg/kg (high-dose group) and 8.2 months for docetaxel with an HR of 0.50 (95% CI, 0.36 to 0.70).

Based on the above report, atezolizumab monotherapy in previously treated patients with NSCLC with TPS ≥ 50% as determined by the 22C3 antibody is estimated to be as effective as pembrolizumab monotherapy.

For a first-line treatment with atezolizumab, a Phase III study (IMpower110) to compare atezolizumab alone vs. platinum-based combination therapy in treatment-naïve patients with stage IV NSCLC with high PD-L1 expression (TC 2/3 or IC 2/3) as determined by the SP142 antibody, and without EGFR mutation and ALK gene translocation has been undertaken, and a follow-up period is ongoing as of April 2018.

A Phase III study (KEYNOTE-024) to compare pembrolizumab alone vs. platinum-based combination therapy in treatment-naïve patients with stage IV NSCLC with TPS ≥ 50%, and without EGFR mutation and ALK gene translocation, who are the subjects of the present study, has been reported. The HR for PFS, defined as the primary endpoint, was 0.50 (10.3 months vs. 6.0 months; 95% CI, 0.37 to 0.68), and the HR for OS, a secondary endpoint, was 0.60 (not reached the median value in either of the groups). These have demonstrated that pembrolizumab monotherapy significantly prolongs PFS and OS compared with platinum-based combination therapy; thus, pembrolizumab monotherapy has been recommended as standard treatment.

In summary, the high efficacy of pembrolizumab monotherapy in treatment-naïve patients with stage IV with TPS ≥ 50% has been demonstrated. However, there is still room remaining for improvement in observed data such as an ORR and PFS, and thus further improvement in efficacy with new combination therapies is expected.

Bevacizumab, which is selected to be a drug to be administered in combination with atezolizumab in this clinical study, has been used as a standard first-line treatment for

non-squamous NSCLC for a long time. For a main clinical study of bevacizumab in patients with non-squamous NSCLC, Phase III studies (E4599 study[8)] and AVAiL Study[9)]) in treatment-naïve patients with advanced or recurrent non-squamous NSCLC revealed the additional effect of bevacizumab to platinum-based combination therapy which was conventional standard treatment. In E4599 study, bevacizumab monotherapy was continued until disease progression in patients without disease progression after 6 cycles of treatment with bevacizumab, carboplatin and paclitaxel. A total of 878 patients were enrolled. Median OS was 12.3 months and 10.3 months (HR = 0.79; p < 0.003) in the bevacizumab combination group and chemotherapy group, respectively, median PFS was 6.2 months and 4.5 months, respectively (HR = 0.66; p < 0.001), and the ORR was 35% (133/381) and 15% (59/392), respectively (p < 0.001). AVAiL Study (BO17704) was a multicenter, double-blind, controlled, Phase III study to compare bevacizumab + cisplatin + gemcitabine vs. placebo + cisplatin + gemcitabine as a first-line treatment in patients with advanced or recurrent non-squamous NSCLC. A total of 1043 patients were enrolled. Bevacizumab combination therapy until disease progression reduced the risk of disease progression. The HR for PFS in the bevacizumab 7.5 mg/kg group was 0.75 (median PFS, 6.7 months vs. 6.1 months; p = 0.003), and the HR for PFS in the bevacizumab 15 mg/kg group was 0.82 (median PFS, 6.5 months vs. 6.1 months; p = 0.03). These results were maintained during longer follow-up. OS was a secondary endpoint, but clinical benefit based on the PFS was not linked to a significant benefit in terms of OS. Median OS in this study was still more than 13 months in all the treatment groups.

A randomized Phase II study (JO25567) to investigate the additional effect of bevacizumab to erlotinib, a molecular-targeted drug and EGFR inhibitor, in patients with EGFR mutation-positive NSCLC suggested an effect of bevacizumab to prolong PFS when it was added to erlotinib.

For bevacizumab, VEGF inhibitor (e.g., bevacizumab) in combination with an immune checkpoint inhibitor (atezolizumab) selected for this clinical study, several reports have provided scientific evidence supporting VEGF combination in basic and clinical studies. Bevacizumab has been reported to possibly induce immune response via various mechanisms such as an increase in T cell migration in tumors by trapping the VEGF in tumor environment, decrease in the frequency of myeloid-derived suppressor cell (MDSC) induction, reductions in inhibitory cytokines, tumor-infiltrating regulatory T cells and MDSC, and augmentation in CD8+ and CD4+ central memory T cells. Furthermore, several reports have documented recent findings on a VEGF inhibitor (e.g., bevacizumab) in combination with an immune checkpoint inhibitor supporting scientific evidence in clinical studies. The results of a Phase I study (cohort study) of pembrolizumab + ramucirumab, an anti-VEGFR2 antibody, in patients who received second- to fourth-line treatments reported that the ORR was 29.6% (95% CI,

13.7% to 50.2%)[19]. Also, the marked therapeutic response to bevacizumab in combination with atezolizumab in patients with metastatic renal cell carcinoma (RCC) has been reported (Sznol et al. 2015).

For a clinical study evaluating atezolizumab + bevacizumab therapy, a global randomized Phase III study (IMmotion150) to investigate the efficacy of atezolizumab + bevacizumab therapy in treatment-naïve patients with locally advanced or metastatic renal cancer was reported and showed significant prolongation of PFS that median PFS for patients with PD-L1 ≥ 1% was 14.7 months in the atezolizumab + bevacizumab group as compared with 5.5 months in the atezolizumab group. Furthermore, the results of the expression of PD-L1 IC as determined by the SP142 antibody suggested higher efficacy in patients with high PD-L1 expression (IC ≥ 10%). The ORR was 46% and complete response rate was 12% for atezolizumab + bevacizumab therapy as compared with the ORR of 26% for atezolizumab monotherapy.

For lung cancer, a randomized Phase III study to compare 3 groups, carboplatin + paclitaxel + bevacizumab vs. carboplatin + paclitaxel + atezolizumab vs. carboplatin + paclitaxel + bevacizumab + atezolizumab in treatment-naïve patients with advanced or recurrent non-squamous NSCLC has been undertaken. The results of an analysis comparing PFS between carboplatin + paclitaxel + bevacizumab vs. carboplatin + paclitaxel + bevacizumab + atezolizumab have been reported so far. The additional effect of atezolizumab has been demonstrated: Median PFS was 6.3 months for carboplatin + paclitaxel + bevacizumab, a conventional standard treatment, and 8.3 months for a study group, carboplatin + paclitaxel + bevacizumab + atezolizumab, with an HR of 0.505 (95% CI, 0.377 to 0.675). Particularly, the HR tended to be low in a group with high PD-L1 expression (PD-L1 TC ≥ 50%; IC ≥ 10%) as determined by staining with Ventana SP142 antibody (HR = 0.39; 95% CI, not disclosed). In this study, AEs associated with the addition of atezolizumab to carboplatin + paclitaxel + bevacizumab were reported. Grades 3 to 4 toxicities increased due to the addition of atezolizumab were hepatitis in 5% of the patients in the group with atezolizumab (1% in the groups without atezolizumab), pneumonitis in 2% (vs. 1%) and pancreatitis in 1% (vs. 0%); thus, there was no large increase in AEs due to the addition of atezolizumab. Hence, atezolizumab + bevacizumab therapy without carboplatin + paclitaxel is determined to be tolerable treatment.

Based on these reported results of clinical and basic studies, atezolizumab + bevacizumab therapy for patients with non-squamous NSCLC with high PD-L1 expression (TPS ≥ 50%) may enhance antitumor immune response and improve clinical benefits so that sufficient rationales for its evaluation are found to be available.

The present study will investigate the efficacy and safety of atezolizumab + bevacizumab therapy in treatment-naïve patients with advanced or recurrent

non-squamous NSCLC with high PD-L1 expression (TPS ≥ 50%, 22C3 antibody) and without EGFR mutation or ALK gene translocation and ROS1 gene translocation and evaluate whether or not this combination therapy is a suitable treatment to be selected as a study treatment group in a Phase III study as compared with historical data on pembrolizumab monotherapy.

2.4. Study Design

2.4.1. Clinical hypothesis in the study and phase setting

This is a multicenter, single-arm Phase II study to examine the efficacy and safety of atezolizumab + bevacizumab therapy. If this study demonstrates the efficacy of atezolizumab + bevacizumab therapy, a Phase III study to evaluate the efficacy of pembrolizumab alone vs. atezolizumab + bevacizumab therapy will be planned.

2.4.2. Rationales for the selection of endpoints

・Primary endpoint:

The effect of atezolizumab + bevacizumab will be examined by defining an ORR as an endpoint. Results by the central assessment will be adopted because a bias for ORR by researchers appears.

Secondary endpoints:

For efficacy endpoints for the study treatment, not only investigator-assessed PFS but also DOR, OS and safety will be chosen to be secondary endpoints.

2.4.3. Rationale for the determination of the number of subjects to be enrolled

When setting a threshold ORR of 40% and expected ORR of 62%, the number of subjects necessary for evaluation based on an exact binomial distribution under conditions of a one-sided significance level of 5% and power of 80% is 38 subjects.

[Rationales for setting the threshold value and expected value]:

The ORR for pembrolizumab monotherapy in treatment-naïve patients with NSCLC with high PD-L1 expression (TPS ≥ 50%) was reported to be 44.8% (95% CI, 36.8% to 53.0%)[2)]. Based on this result, the threshold ORR was set to 40%. The expected ORR was set to 62% in expectation of additional 22%, which is a clinically meaningful value, because high response could be anticipated for patients with high PD-L1 expression based on the following report and in consideration of its use as a first-line treatment: The ORR for atezolizumab in patients who previously received a second- or third-line treatment was 13.6% (95% CI, 10.5% to 17.3%) while they did not have high PD-L1 expression; and the ORR in a Phase I study of pembrolizumab + ramucirumab therapy

in patients who previously received a second- to fourth-line was 29.6% (95% CI, 13.7% to 50.2%)[19)].

2.5. Summary of Anticipated Benefits and Disadvantages Associated with Participation in the Study

Pembrolizumab monotherapy is a standard treatment for non-squamous NSCLC with high PD-L1 expression (TPS ≥ 50%) as determined by immunohistochemical staining using Dako 22C3 antibody, a companion diagnostic for pembrolizumab, and without driver mutation-positive NSCLC such as EGFR mutation-positive, ALK translocation-positive and ROS1 translocation-positive to which molecular-targeted drugs should be prioritized. Nonetheless, atezolizumab monotherapy is also expected to exert an effect to prolong survival equivalent to that of pembrolizumab monotherapy because, as previously mentioned, the results of a Phase III study (OAK) in previously treated patients with NSCLC and the results of an efficacy analysis, which was retrospectively performed using the 22C3 antibody, revealed the effect of atezolizumab to prolong survival equivalent to an HR in the high-dose pembrolizumab (10 mg/kg) group vs. the docetaxel group for the subgroup with TPS ≥ 50%. Hence, there may be no significant demerit of using atezolizumab in the target study population in terms of the efficacy. In addition, the results of a Phase III study of atezolizumab + bevacizumab + platinum-based combination therapy have been disclosed, and thus the administration of atezolizumab + bevacizumab therapy is found to cause no significant disadvantage from the viewpoint of toxicity. Consequently, to evaluate the efficacy and safety of atezolizumab + bevacizumab therapy as a first-line treatment for non-squamous NSCLC in a prospective clinical study is considered to be not only scientifically but also ethically appropriate.

2.6. Planned Number of Subjects to Be Enrolled and Study Period

Planned number of subjects to be enrolled: 38 subjects

Study period: Three years from August 1, 2018 to July 31, 2021

Enrollment period: One year from August 1, 2018 to July 31, 2019

Follow-up period: Two years from the day of the enrollment of the last subject

3. CRITERIA AND DEFINITIONS USED IN THE STUDY

3.1. Definition of Period

Periods used in the study are defined as follows:

Study period: From the start day of enrollment to the completion of the follow-up

period

Enrollment period: From the start day of enrollment to the end day of enrollment

Follow-up period: Two years from the end day of enrollment

3.2. Definition of Pathological Diagnosis

As specified in the World Health Organization (WHO) classification, the fourth edition.

3.3. Definition of Stage Classification

TNM Classification (UICC 8th Edition) will be utilized for staging at the initial diagnosis of NSCLC.

3.4. Definition of Response Assessment

For assessments of the antitumor activity, the primary endpoint, and PFS, a secondary endpoint, the “New response evaluation criteria in solid tumors: Revised RECIST guideline (version 1.1) - Japanese translation version by the Japan Clinical Oncology Group (JCOG) - (RECIST v1.1 Japanese translation by the JCOG)” will be used.

3.5. Definition of Adverse Event

AEs and reactions will be assessed in accordance with the “National Cancer Institute-Common Terminology Criteria for Adverse Events (NCI-CTCAE) Version 4.0, Japanese translation version by the JCOG.”

An AE is any unfavorable and unintended sign (including an abnormal laboratory finding), symptom or disease observed during treatment or therapeutic procedures, whether or not considered related to the treatment or therapeutic procedures.

4. SELECTION OF THE SUBJECTS

4.1. Inclusion Criteria

Patients, who meet all of the following criteria, will be included in the study:
[Informed consent and age]

1) Patients who have personally provided written consent after receiving an adequate explanation on the contents of the clinical study prior to enrollment in the study.
2) Patients aged ≥ 18 to ≤ 75 years at the time of providing consent

[Tumor histologic type and marker]

3) Patients with a histologically confirmed diagnosis of non-squamous NSCLC
4) Patients with the high PD-L1 expression (TPS ≥ 50%, Dako 22C3 antibody) in tumor tissue by immunohistochemistry using archived tumor tissue samples

collected in the past or tissue samples taken by a biopsy at screening

5) Patients who are confirmed to be negative for EGFR mutation, ALK gene translocation and ROS1 gene translocation

[Status and spread of the lesion]

6) Patients with stage III and stage IV NSCLC not treatable with radical radiotherapy or postoperative recurrent NSCLC
7) Patients with measurable disease as defined by the RECIST version 1.1. However, the site of radiation is not required to be a measurable lesion.
8) Patients without hemoptysis risk factors associated with bevacizumab such as macrovascular infiltration, direct tumor penetration into the trachea or bronchus and evident tumor necrosis and cavitation
   a) Absence of clear tumor infiltration into a major thoracic blood vessel on other images
   b) Absence of evident cavitation of the lung lesion on images
9) Absence of superior vena cava syndrome
10) Absence of spinal cord compression symptoms
11) Absence of evident deep vein thrombosis or arterial thrombosis on images
12) Absence of brain metastasis that is symptomatic or requires antiedematous agents such as steroid or anticonvulsants for controlling symptoms
13) Absence of pleural effusion, ascites or cardiac effusion requiring drainage (Patients with stable symptoms after drainage are eligible.)

[Previous treatment, tests and procedures]

14) Patients who were not previously treated with either chemotherapy or radiotherapy including treatment for other cancer types. However, patients with recurrent lung cancer at least 6 months after postoperative adjuvant chemotherapy are eligible.
15) Patients who were not previously treated with radiotherapy for the primary lesion. However, patients who previously received radiotherapy for metastases, excluding chest irradiation, for palliative treatment are eligible.
16) The following length of time must be elapsed from the end of prior treatments or procedures to the time of enrollment (The same day of a week is acceptable):
   a) Surgery (including exploratory or diagnostic thoracotomy and resection of metastatic brain tumors) 4 weeks
      Thoracoscopic pleural biopsy 2 weeks
   b) Palliative radiotherapy for pain alleviation (excluding chest irradiation) 2 weeks
   c) Thoracic cavity drainage for carcinomatous pleurisy 2 weeks
   d) Biopsy with resection and procedure for trauma (excluding patients with untreated wounds) 2 weeks
   e) Blood transfusion and administration of hematopoietic factors 2 weeks

[General condition and laboratory test]

17) Patients with Eastern Cooperative Oncology Group Performance Status (ECOG PS) 0 or 1
18) Patients without severe impairment of major organs and who meet criteria listed below. The most recent data within 14 days from the date of enrollment should be used for enrollment (Data on the date 2 weeks before the date of enrollment, which is the same day of a week, are acceptable.).

Neutrophil count:	≥ 1500/mm^3
Hemoglobin:	≥ 9.0 g/dL
Platelet count:	≥ 10.0×10,000/mm^3
International normalized ratio of prothrombin time (PT-INR):	≤ 1.5
Aspartate aminotransferase (AST):	≤ 100 IU/L
Alanine aminotransferase (ALT):	≤ 100 IU/L
Total bilirubin:	≤ 1.5 mg/dL
Creatinine:	≤ 1.5 mg/dL
Percutaneous oxygen saturation (SpO_2):	≥ 90% (room air)
Urine protein:	≤ 1+

4.2. Exclusion Criteria

Patients who meet any of the following criteria will be excluded from the study:
[Double cancer]

1) Patients with active double cancer*1
   *1: The term, double cancer, refers to simultaneous double cancer and metachronous double cancer with a disease-free period of ≤ 5 years. Carcinoma in situ or lesions equivalent to intramucosal carcinoma, which are judged to have cured by local therapy, are not included in active double cancer.

[Complication: Bleeding]

2) Patients with a past or current history of hemoptysis (bleeding of approximately ≥ 2.5 cc per episode from the respiratory organ) or the following bloody sputum:
   a) Persistently (≥ 1 week) appearing bloody sputum
   b) Bloody sputum that was continuously treated for ≤ one month or requires continuous treatment with oral hemostatic agents
   c) Bloody sputum that was treated or requires treatment with hemostatic agents for injection
3) Presence of a bleeding tendency (coagulation disorder)
4) Patients who received antithrombotic drugs (excluding low-dose aspirin) within 10 days or require treatment with them during the study period

[Complication: Infection]

5) Patients with local infection or systemic active infection requiring surgical procedures such as drainage
6) Patients with active type B or C hepatitis (Patients who are hepatitis B surface [HBs] antibody-positive are eligible if a viral load is below detection and hepatitis is not active.)

[Complication: Interstitial pneumonia and autoimmune disease]

7) Patients with a past or current history of interstitial lung disease evident on computed tomography (CT) scans, drug-induced lung disorder and pneumonitis requiring steroid treatment
8) Patients with a past or current history of autoimmune disease and autoimmune disease that required steroid therapy
9) Patients who require continuous systemic treatment (oral or IV) with steroid at a dose higher than a prednisolone equivalent dose of 10 mg/day and patients who are on immunosuppressive drugs. Patients, who are on oral steroid equivalent to a prednisolone dose of ≤ 10 mg/day as replacement therapy for adrenal

insufficiency, etc., are eligible.

[Complication: Cardiovascular disease]

10) Patients with cerebrovascular disorder within one year. Patients with persistent cerebral ischemia attack
11) Patients with symptomatic congestive cardiac failure or unstable angina pectoris, or a past history of myocardial infarction within one year before enrollment
12) Patients who are concomitantly using drugs that are known to cause clinically serious arrhythmia (complete left bundle branch block, and third-degree or second-degree degree atrioventricular block) with QTc > 470 ms on electrocardiogram and QTc interval prolongation
13) Patients with a past history within one year of or concurrent digestive tract perforation, fistula or diverticulitis

[Complication: Others]

14) Untreated bone fracture (excluding compressed fracture associated with osteoporosis) or severe wounds
15) Uncontrollable peptic ulcer
16) Hypertension (defined as systolic blood pressure of ≥ 150 mmHg or diastolic blood pressure of ≥ 100 mmHg observed for several days) uncontrollable with standard drug therapy

[Other]

17) Patients with a history of serious hypersensitivity or with hypersensitivity to the ingredients or additives of atezolizumab and bevacizumab
18) Pregnant women, lactating women, women who may be pregnant at present or patients not intending to practice contraception
19) Patients who are judged to be difficult for enrollment in the study due to clinically significant mental disease
20) Patients who are determined by the investigator/subinvestigator to be not suitable due to other reasons

5. SUBJECT ENROLLMENT

5.1. Enrollment Procedures

The investigator/subinvestigator or study collaborator should give an explanation about the study to candidate subjects and receive their written consent.

The investigator/subinvestigator or study collaborator should verify that the subjects meet all the inclusion criteria and none of the exclusion criteria, and then register them using the electronic data capture (EDC) system. After the subjects are reverified to be eligible on the EDC system, they should be enrolled in the study.

The investigator/subinvestigator or study collaborator should check enrollment numbers on the EDC system.

Registration using the EDC system:

- The investigator/subinvestigator or study collaborator should access to the web registration system for the study via the Internet.
- To enter necessary information in accordance with instructions in the enrollment system to make registration.
- To obtain an enrollment number as a registration result.
- Registration may be accepted 24 hours excluding time of maintenance.

5.2. Points to Note

1) Once subjects are registered, their registration will not be cancelled (They will not be deleted from the database.).
2) In the case of double registration, the first registration information (enrollment number) will be adopted as a rule.
3) Error or double registration should be promptly notified if identified.
4) In principle, safety should be confirmed within 7 days from the day of enrollment (The same day of a week is acceptable.), and then the protocol treatment should be initiated.

5.3. Contact Person for Enrollment

Clinical Study Coordinating Secretariat

(IQVIA Services Japan K.K.?)

Mitsuru Shimomura

Tel: 080-3024-6919 Fax: 03-6859-9851

E-mail: WJOG10718L@iqvia.com

6. PROTOCOL TREATMENT PLAN

6.1. Investigational Drug and Drug for the Clinical Study

Atezolizumab and bevacizumab will be supplied by Chugai Pharmaceutical Co., Ltd. as the investigational drug and the drug for the clinical study, respectively.

6.1.1. Properties of the investigational drug and the drug for the clinical study

**Nonproprietary name**	**Abbreviation**	**Dosage form and content**	**Manufacturer**
Atezolizumab	MPDL3280A	1200 mg injection solution	Chugai Pharmaceutical Co., Ltd.
Bevacizumab	RO4876646	400 mg/16 mL injection solution	Chugai Pharmaceutical Co., Ltd.

6.1.2. Packaging and labels of the investigational drug and the drug for the clinical study

Labeling and packaging are presented in the separately specified "Procedures for the management of the investigational drug and the drug for the clinical study."

6.1.3. Storage of the investigational drug and the drug for the clinical study

The investigational drug and the drug for the clinical study should be stored in a safe place under appropriate storage conditions. The appropriate storage conditions and transport conditions are presented on the labels attached to the boxes of the investigational drug and the drug for the clinical study, the Procedures for the management of the investigational drug and the drug for the clinical study, and the investigator's brochure.

6.1.4. Management of the investigational drug

The investigational drug will be provided from the investigational drug supplier to the sponsor-investigator at each study site through the coordinating investigator at a specified time point after the submission of the notification of a clinical study protocol. Specific procedures for investigational drug supply will be stipulated in separately issued "Procedures for investigational drug management." The sponsor-investigator should explain the contents of the clinical study to the investigational drug manager at his/her affiliated study site, submit the "Procedures for investigational drug management" and request him/her for the storage and control of the investigational drug. The investigational drug manager should properly store and control the investigational drug and outer boxes during the study period regardless of the use of the investigational drug and prepare an investigational drug management chart to understand the use status of the investigational drug. The sponsor-investigator should check consistency of the investigational drug management records, remaining drugs and information documented in the case report forms (CRFs), immediately perform an investigation into the cause if inconsistencies are found, and make necessary corrections.

After the completion of the clinical study, the investigational drug manager should return unused investigational drug to the sponsor-investigator. When returning it, matters concerning the subjects' privacy such as their names (initials) and medical chart identifications (IDs) should be masked. When unused investigational drug is lost, its details and reasons should be recorded. The sponsor-investigator should discard all unused investigational drug that is returned from the investigational drug manager.

6.2. Protocol Treatment

6.2.1. Atezolizumab + bevacizumab therapy

Atezolizumab at a fixed dose of 1200 mg (equivalent to a mean dose of 15 mg/kg per body weight) has been chosen based on nonclinical study data and clinical data obtained from PCD4989g study. The subjects will receive atezolizumab 1200 mg as an IV infusion once every 21 days in adequate facilities and drug arrangement under the supervision of trained personnel so that reactions, which may be serious, can be immediately handled. For detailed information on dosing, see the investigator's brochure for atezolizumab.

At the time of dosing, atezolizumab should be administered first followed by bevacizumab administered at an interval of at least 5 minutes.

The dose of bevacizumab is 15 mg/kg and administered by IV infusion once every 21 days on Day 1 of each cycle. For the start day of each cycle, + 7 days (up to the same day of a week of the planned treatment initiation) will be acceptable. The same dose of bevacizumab should be used throughout the study period unless a change in body weight from baseline does not exceed more than 10% based on body weight at the time of enrollment. It is unnecessary to modify the dose based on actual body weight except for the case where such is necessary in accordance with guidelines or criteria used at each site. For detailed information on dosing, see the package insert of bevacizumab.

The maximum duration of treatment will be 2 years. For subjects who can receive the therapy for ≥ 2 years and wish to continue it, however, their handling should be individually discussed with the clinical study coordinating committee.

6.2.2. Administration of atezolizumab

6.2.2.1. Initial dose

- During the first dosing, atezolizumab should be administered as paying careful attention to infusion reactions (Vital signs, etc. should be checked before dosing, and 15, 30, 45 and 60 minutes after the start of infusion.).
- No premedication will be given.
- Atezolizumab (The contents of one vial should be dissolved in 250 mL of 0.9% normal saline) should be administered over 60 minutes (± 15 minutes).
- The subjects should be explained that late-onset infusion reactions may occur and advised to consult the investigator/subinvestigator if symptoms appear.

6.2.2.2. Second and subsequent doses

- From the second and subsequent doses, an antihistamine may be administered as a

premedication to subjects, who developed infusion reactions during the initial infusion, at the discretion of the investigator/subinvestigator.

- If tolerability was good and no infusion reaction-related AEs occurred during the initial infusion, the second infusion may be given for 30 minutes (± 10 minutes).
- For subjects who developed infusion reactions during the previous dosing, the subsequent infusion should be administered over 60 minutes (± 15 minutes).
- If no infusion reaction appeared, the subsequent doses should be administered for 30 minutes (± 10 minutes) as paying careful attention to vital signs.

6.2.3. Administration of bevacizumab

6.2.3.1. Initial dose

- Bevacizumab (15 mg/kg should be dissolved in 100 mL of 0.9% normal saline. It is acceptable to make a total of 100 mL in accordance with a standard at the site in the case, for example, where the total dose is large.) should be administered over 90 minutes (± 15 minutes).
- For the initial dose, attention should be paid to infusion reactions during a period from the start of infusion to at least 2 hours postdose.

6.2.3.2. Second and subsequent doses

- If tolerability was good and no infusion reaction occurred during the initial infusion, the second infusion may be given for 60 minutes (± 10 minutes). If the 60-minute infusion is well tolerated, all the subsequent doses may be administered for 30 minutes (± 10 minutes).
- For the second and subsequent doses, attention should be paid to infusion reactions over at least one hour postdose.
- If infusion reactions occurred, an antihistamine may be administered as a premedication from the next dose, but the duration of bevacizumab infusion shall not be reduced for that dose.
- If the next dose is well tolerated as a result of the premedication, the duration of the subsequent infusion may be reduced by 30 minutes as long as the premedication is continued.
- If infusion reactions occurred at the second dose given for 60 minutes, all the subsequent doses should be administered for 90 minutes (± 15 minutes).
- Similarly, if infusion reactions occurred during the second infusion administered for 30 minutes, all the subsequent doses should be administered for 60 minutes (± 10 minutes).

6.2.4. Continuation of treatment with a single drug

Either one of atezolizumab or bevacizumab may be discontinued or interrupted (e.g.,

discontinuation or treatment interruption due to AEs), and monotherapy may be continued for a period during which disease progression is clinically controllable and AEs are manageable or for 2 years after the start of the initial dose. For subjects who can receive the therapy for ≥ 2 years and wish to continue it, their handling should be individually discussed with the clinical study coordinating committee.

6.3. Treatment Initiation

In principle, safety should be confirmed within 7 days from the day of enrollment (The same day of a week is acceptable.), and then the protocol treatment should be initiated.

When the protocol treatment is started more than 7 days after the day of enrollment, the reasons should be reported.

6.3.1. Withdrawal before treatment

When it is determined that the protocol treatment cannot be started for some reason, the subject should be withdrawn from the study before the protocol treatment, the details should be provided in a report of treatment completion, and the report should be promptly submitted.

6.4. Completion of the Protocol Treatment

1) When disease progression is found to be not clinically controllable with the protocol treatment
2) When the protocol treatment cannot be continued due to AEs:
   (i) When Grade 4 AEs occurred;
   (ii) When Grade ≥ 2 interstitial lung disease/pneumonitis occurred;
   (iii) When AEs cannot be controlled despite treatment with hormone replacement therapy or insulin supplementation; and
   (iv) When the investigator/subinvestigator determined that the protocol treatment cannot be continued in consideration of safety.
3) When the subjects requested to discontinue the protocol treatment:
   (i) When the subjects requested discontinuation due to reasons related to AEs;
   (ii) When the subjects requested discontinuation due to reasons not related to AEs; and
   (iii) When the subjects withdrew their consent (see the Section 6.4.4).
4) When the subjects died during the protocol treatment
5) When the subjects are found to be ineligible after enrollment, and the

continuation of the protocol treatment is determined to be not beneficial for them

6) When it is judged that the protocol treatment cannot be continued due to transfer to another hospital for some reason during the protocol treatment period.

6.4.1. Definition of the completion of the protocol treatment

When the protocol treatment can be administered for 2 years, it is deemed as being completed because in this study, after progression is confirmed during the protocol treatment, the protocol treatment can be still continued until disease progression is determined to be clinically not controllable with it.

6.4.2. Ineffectiveness of the protocol treatment

For reasons for the completion of the protocol treatment, clinical judgment should be prioritized for “disease progression being not controllable.”

6.4.3. Definitions of AEs when completing the protocol treatment

The following adverse reactions (hematologic toxicities) in the CTCAE Version 4.0 will be excluded:
“Anemia,” “bone marrow hypocellular,” “lymphocyte count decreased,” “neutrophil count decreased,” “white blood cell decreased,” “platelet count decreased” and “CD4 lymphocytes decreased”
The onset of Grade 4 biochemical values, which do not require treatment and are determined to be not life-threatening, is not included in requirements for treatment discontinuation.

6.4.4. Precautions for withdrawal of consent

No data after the withdrawal of consent should be collected. The subjects’ intention as to whether it is refusal of the protocol treatment and/or tests or truly withdrawal of consent should be thoroughly verified.

6.5. Criteria for Administration of Each Cycle (Course) and Criteria for Treatment Changes

A criterion for treatment discontinuation, interruption and skip is AEs.

Terms used in criteria for administration and criteria for treatment changes are as follows:

Discontinuation:	Premature termination of part or all of treatment without resuming
Treatment interruption:	Temporary discontinuation with possibility of resuming
Skip:	Moving on to the next schedule without administration of

part or all of treatment

6.5.1. Criteria for the start of each cycle (course)

It should be confirmed that criteria for the start of each cycle (course) are met, and then each cycle (course) should be initiated (regardless of atezolizumab + bevacizumab therapy or monotherapy with either one of them).

6.5.2. Criteria for administration of atezolizumab in Cycle (Course) 2 and subsequent cycles (courses)

In this study, the dose of atezolizumab may not be reduced. While bevacizumab treatment is interrupted, atezolizumab may be administered.

From Cycle (Course) 2 and onwards, atezolizumab should be administered after confirming that all of the following criteria are met before infusion on the start day of a cycle or the previous day of infusion:

Table 6.5-1 Criteria for starting atezolizumab in each cycle (course)

When atezolizumab is administered, see the management of AEs unique to it (separate volume).

Endpoint	Criteria for the start of each cycle (course)
(1) PS	0 to 2
(2) Immune-related events (lung, liver, digestive tract, asymptomatic endocrine, eyes, myocarditis, and neuropathy)	Grade ≤ 1
(3) Amylase increased, hyperglycemia and skin events	Grade ≤ 2
(4) Immune-related meningoencephalitis	Grade 0

Even if the above criteria are not met, the therapy may be postponed if the investigator/subinvestigator judges that it should be postponed in consideration of safety.

When the criteria for administration are not fulfilled for more than 7 days, administration of atezolizumab should be skipped. In such a case, if criteria for administration of bevacizumab are satisfied, bevacizumab alone should be administered.

For subjects who have temporarily or permanently discontinued bevacizumab treatment (e.g., due to AEs), atezolizumab treatment may be continued if it is considered to be clinically beneficial.

6.5.3. Criteria for discontinuation of atezolizumab

1) When Table 6.5-1 Criteria for starting atezolizumab in each cycle are not met after therapeutic actions were taken based on the management of AEs unique to it (separate volume) and despite interruption for 105 days.

2) When the AEs listed below occurred.

   See the management of AEs unique to it (separate volume).

Endpoint	Criteria for discontinuation of atezolizumab
(1) Immune-related events (pneumonitis, liver, eyes, myocarditis and neuropathy)	Grade ≥ 3
(2) Immune-related events (diarrhea, colitis, panhypopituitarism, pancreatitis and skin disorder)	Grade ≥ 4
(3) Immune-related meningoencephalitis	Any grade
(4) Allergic reaction	Grade ≥ 3

3) When the investigator/subinvestigator determined that atezolizumab should be

Seto T, *et al. J Immunother Cancer* 2022; 10:e004025. doi: 10.1136/jitc-2021-004025

discontinued.

6.5.4. Criteria for resuming atezolizumab after its interruption

1) In subjects who developed atezolizumab-related AEs requiring interruption, atezolizumab may be temporarily suspended. For subjects who do not meet the criteria for administration of atezolizumab for more than 105 days from the planned day of the next infusion even after therapeutic actions were taken based on the management of AEs unique to it (separate volume), atezolizumab treatment should be discontinued. When the criteria for administration of atezolizumab are fulfilled after an interruption period of more than 105 days, and the investigator/subinvestigator determined that its reintroduction may be beneficial for the subjects, the investigational treatment may be resumed upon discussion with the coordinating investigator.
2) In subjects requiring the tapering of steroid used for the treatment of AEs, the investigational drug may be interrupted for more than 105 days until steroid is discontinued or the dose is reduced to a prednisone equivalent dose of ≤ 10 mg/day. The acceptable duration of interruption should be decided upon agreement between the investigator/subinvestigator and coordinating investigator.
3) Treatment interruption due to reasons other than AEs (e.g., surgical treatment) and the acceptable duration of interruption should be determined by the investigator/subinvestigator upon discussion with the coordinating investigator.

6.5.5. Criteria for administration of bevacizumab in Cycle (Course) 2 and subsequent cycles (courses)

In this study, the dose of bevacizumab may not be reduced. While atezolizumab treatment is interrupted, bevacizumab may be administered.

From Cycle (Course) 2 and onwards, bevacizumab should be administered after confirming that all of the following criteria are met before infusion on the start day of a cycle or the previous day of infusion:

Table 6.5-2 Criteria for starting bevacizumab in each cycle (course)

Endpoint	Criteria for the start of each cycle (course)
(1) PS	0 to 2
(2) Proteinuria	Grade ≤ 1
(3) Thromboembolic event	Grade ≤ 1
(4) Bronchopulmonary hemorrhage	Grade 0
(5) Hemorrhage (excluding bronchial hemorrhage)	Grade ≤ 1
(6) Hypertension	Systolic blood pressure of ≤ 150 mmHg and diastolic blood pressure of ≤ 100 mmHg regardless of oral treatment

Even if the above criteria are not met, the therapy may be postponed if the investigator/subinvestigator judges that it should be postponed in consideration of safety.

When the criteria for administration are not fulfilled for more than 7 days, administration of bevacizumab should be skipped. In such a case, if criteria for administration of atezolizumab are satisfied, atezolizumab alone should be administered.

For subjects who have temporarily or permanently discontinued atezolizumab treatment (e.g., due to AEs), bevacizumab treatment may be continued during a period when disease progression can be clinically controlled if it is considered to be clinically beneficial.

6.5.6 Criteria for discontinuation of bevacizumab

Bevacizumab should be discontinued when any of the following items is noted. In such a case, treatment with atezolizumab alone should be continued in accordance with the Section 6.2.4:

1) Table 6.5-2 Criteria for starting bevacizumab in each cycle (course) are not satisfied despite interruption for 42 days.

2) When the following AEs occurred:

Endpoint	Criteria for discontinuation of bevacizumab
(1) Thromboembolic event	Grade ≥ 2
(2) Bronchopulmonary hemorrhage	Grade ≥ 2
(3) Hemorrhage (excluding bronchial hemorrhage)	Grade ≥ 3
(4) Digestive tract perforation	Any grade
(5) Allergic reaction	Grade ≥ 3
(6) Myocardial infarction, ventricular arrhythmia and heart failure	Grade ≥ 3
(7) Others	Hypertension (hypertensive crisis or hypertensive encephalopathy) and severe proteinuria (nephrosis)

3) When the investigator/subinvestigator determined that bevacizumab should be discontinued.

6.6. Concomitant Therapies and Supportive Therapies

6.6.1 Specified concomitant therapies and supportive therapies

In this study, there are no particularly specified concomitant therapies or supportive therapies.

•6.6.2 Permitted concomitant therapies and supportive therapies

From Cycle 2, treatment with an antihistamine may be given before administration of atezolizumab.

During the study period, the following therapies should be continued:

- Hormone replacement therapy
- Prophylactic anticoagulant therapy (≤ 324 mg/day of aspirin)
- When palliative radiotherapy excluding chest irradiation (e.g., palliative treatment for known bone metastasis) does not affect assessment of tumors or lesions (e.g., when tumors are not inevaluable in accordance with the RECIST v1.1 or the site of irradiation is not only that lesion), it is unnecessary to interrupt treatment with atezolizumab and bevacizumab during palliative radiotherapy

- Inactivated influenza vaccine
- Steroid (oral prednisone equivalent dose of ≤ 10 mg) for chronic obstructive pulmonary disease
- Mineral corticoid (e.g., fludrocortisone)
- Low-dose steroid for subjects with orthostatic hypotension or adrenocortical insufficiency

The investigator/subinvestigator should, in principle, manage the subjects with supportive therapy in accordance with clinical needs or regional standard treatment. Subjects who developed infusion reactions may be treated with symptomatic treatment such as acetaminophen, ibuprofen, diphenhydramine, famotidine, and other H2 receptor antagonists in accordance with standard diagnostic procedures. Serious infusion reactions (e.g., dyspnea, hypotension, wheezing, bronchospasm, tachycardia, decreased oxygen saturation and respiratory distress) should be treated with clinically applicable supportive therapies (e.g., oxygen supplementation and β2-adrenergic agents).

6.6.3 Prohibited concomitant therapies and supportive therapies

- During the study period, no other investigational treatment will be permitted.
- Any combination therapy (whether or not approved by the regulatory authority) for cancer treatment will be prohibited during the period of the investigational treatment (until discontinuation of the investigational drug).

6.7. Subsequent Treatment

In principle, after the completion of the protocol treatment, no treatment for the primary disease will be given until the progression of the primary disease is confirmed. However, this is not applicable to the case of prioritizing the subjects' wish and benefits. When subsequent treatment is administered, its contents will not be specified.

7 ENDPOINTS AND LABORATORY TESTS

7.1 Screening Test and Endpoints

Tests and evaluations listed in the Items 1) to 4) should be carried out before enrollment. If the results of tests performed within the specified period are available, the test results before receiving informed consent may be used upon acceptance of the subjects, and it is not necessary to conduct the tests again after receiving informed consent.

1) Basic subject information

Seto T, *et al. J Immunother Cancer* 2022; 10:e004025. doi: 10.1136/jitc-2021-004025

a) Subject ID code
b) Initials (can be substituted with "* [asterisk]")
c) Date of birth (can be substituted with "* [asterisk]")/age
d) Date of informed consent
e) Gender
f) PS
g) Body height and weight
h) Main medical history (if the subject has a history of malignancies, the date of the last treatment and the contents of treatment)
i) Main complications
j) Presence or absence of drug allergy
k) Tumor histologic type (adenocarcinoma, large cell cancer or others)
l) Diagnostic method (tissue diagnosis, cytologic diagnosis and date of definitive diagnosis)
m) Stage (at enrollment)
n) History of surgery for the primary disease (the contents of treatment [including exploratory thoracotomy and video-assisted thoracic surgery] and the date of the last surgery)
o) History of radiotherapy (the contents of treatment and the date of the last irradiation)
p) History of smoking habit (duration [year] and number of cigarettes)
q) Target and non-target lesions

2) Tests to be performed within one year before enrollment (retest if the disease recurred more than one year post-surgery)

Hepatitis B surface (HBs) antigen, HBs antibody, hepatitis B core (HBc) antibody, hepatitis C virus (HCV) antibody

EGFR gene testing, ALK gene testing and ROS1 gene testing

3) Tests to be carried out within 28 days before enrollment (The same day of a week as the day of enrollment is acceptable.)

a) Chest radiography
b) Thoracic and upper abdominal contrast-enhanced CT scans
c) Contrast-enhanced brain CT scans or contrast-enhanced brain magnetic resonance imaging (MRI)
d) Bone scintigraphy (Bone scintigraphy is unnecessary if fluorodeoxyglucose positron emission tomography [FDG-PET] is conducted.)
e) Diabetes mellitus test, thyroid function test and pituitary and adrenal function tests: (fasting blood glucose, hemoglobin A1c [HbA1c],

thyroid-stimulating hormone [TSH], free triiodothyronine [FT3] or free thyroxine [FT4] or both and adrenocorticotropic hormone [ACTH])

4) Observation and tests to be implemented within 14 days before enrollment (The same day of a week as the day of enrollment is acceptable.)

   a) PS
   b) Subjective and objective symptoms
      - General disorders: Pyrexia and fatigue
      - Skin and subcutaneous tissue disorders: Pruritus, leukoderma and alopecia
      - Gastrointestinal disorders: Constipation, diarrhea, nausea, vomiting and oral mucositis
      - Metabolism and nutrition disorders: Anorexia
      - Vascular disorders: Hypertension and thromboembolism
      - Respiratory, thoracic and mediastinal disorders: Bronchopulmonary hemorrhage, epistaxis and pneumonitis
   c) Laboratory test
      (i) Hematology: White blood cells, neutrophils, hemoglobin and platelets
      (ii) Blood biochemistry: Albumin (Alb), AST, ALT, creatinine (Cr), total bilirubin (T-Bil), sodium (Na), potassium (K) and chlorine (Cl)
      (iii) Coagulation system: PT-INR
      (iv) Urinalysis: Urine protein
      (v) Blood pressure
      (vi) SpO_2
   d) Resting 12-lead electrocardiography (ECG): QTc (Bazett's correction formula, QTc = observed QT / RR interval$^{1/2}$; or Fridericia's correction formula, QTc = observed QT / RR interval$^{1/3}$)

7.6 Tests and Endpoints during the Protocol Treatment Period

For tests and clinical findings after treatment initiation, data at one month after the day of the last dose of the protocol treatment, and if subsequent treatment is started within one month, data before its initiation should be reported.

7.7.1 Safety endpoints

The following items should be evaluated at the start of a cycle (course) or on the previous day:

   a) PS

b) Body weight
c) Subjective and objective symptoms
   - General disorders: Pyrexia and fatigue
   - Skin and subcutaneous tissue disorders: Pruritus, leukoderma and alopecia
   - Gastrointestinal disorders: Constipation, diarrhea, nausea, vomiting and oral mucositis
   - Metabolism and nutrition disorders: Anorexia
   - Vascular disorders: Hypertension and thromboembolism
   - Respiratory, thoracic and mediastinal disorders: Bronchopulmonary hemorrhage, epistaxis and pneumonitis
d) Laboratory test
   (i) Hematology: White blood cells, neutrophils, hemoglobin and platelets
   (ii) Blood biochemistry: Alb, AST, ALT, Cr, T-Bil, Na, K, Cl and blood glucose
   (iii) Coagulation system: PT-INR
   (iv) Urinalysis: Urine protein, pregnancy test in premenopausal women
   (v) Blood pressure
   (vi) SpO_2
e) Resting 12-lead ECG: QTc (Bazett's correction formula, QTc = observed QT / RR interval$^{1/2}$; or Fridericia's correction formula, QTc = observed QT / RR interval$^{1/3}$)
f) Chest radiography
   It may be omitted for cycles (courses) during which chest CT scans were carried out.

7.7.2 Efficacy endpoints

The following imaging tests should be implemented using the same methods as those for the baseline evaluation. The evaluation should be performed once every 6 weeks (± 1 week) until Week 24 by defining the day of enrollment as the starting date of the computation and once every 9 weeks (± 1 week) from Week 25 and onwards. The baseline evaluation methods shall not be changed; however, if no contrast agent can be administered, evaluation using simple CT scans will be permitted.

When the protocol treatment is discontinued due to reasons other than disease progression, efficacy should be assessed at the abovementioned time points whenever possible.

1) Thoracic and upper abdominal contrast-enhanced CT scans (slice thickness of ≤

5 mm)

2) Contrast-enhanced brain MRI or contrast-enhanced brain CT scans (only when the screening test revealed lesions)
3) Bone scintigraphy or FDG-PET (only when progression is suspected)

7.8 Tests and Endpoints after the Completion of the Protocol Treatment

After the completion or discontinuation of the protocol treatment, tests and evaluations to be implemented before the subject completes a period corresponding to the "follow-up period" specified by the protocol are as described below.

7.8.1 Safety evaluation after the end of treatment

1) Laboratory tests

Laboratory tests will not be particularly specified but will be carried out according to routine medical practice.

7.8.2 Efficacy evaluation after the end of treatment

1) Information on progression
Date of progression, site of progression and diagnostic method
2) Information on clinical ineffectiveness
Day when disease progression is determined to be clinically uncontrollable and assessment criteria
3) Information on survival
Date of last survival confirmed, survival status and cause of death
4) Presence or absence of subsequent treatment (surgery, radiotherapy, name of drug, start day of treatment and PS at the start of treatment). For subjects who received subsequent treatment, the subsequent treatment should be also followed up whenever possible.

7.9 Study Schedule

Table 7.9-1 Clinical study schedule

Item	Before enrollment	Each cycle (once every 3 weeks)	At discontinuation or the time of progression	Follow-up period
Informed consent	●			
Subject background characteristics	●			
EGFR, ALK and ROS1gene testing	●1 year			
HBs antigen, HBs antibody, HBc antibody and HCV antibody	●1 year			
Diabetes mellitus, thyroid and adrenal function tests	●	○		
Physical findings (e.g., PS, body weight and subjective symptoms)	●	●	○	○
Chest radiography	●28	●1)	○	○
Thoracic and abdominal CT 2)	●28	●Every 6 weeks until Week 24<br>●Every 9 weeks from Week 25 and onwards		
Brain CT or MRI 2)	●28	○		
Bone scintigraphy or FDG-PET 2)	●28	○		
Clinical findings	●14	●	●	○
Laboratory tests (hematology and urinalysis)	●14	●	○	○
Blood pressure measurement	●14	●	●	○
SpO_2 measurement	●14	●	●	○
Resting 12-lead ECG	●14			

●: Mandatory

●XX: To be implemented within XX days before enrollment

◎: Optional

○: As specified in routine medical practice

1) Chest CT may be substituted with that performed during the same period.

2) When being discontinued due to reasons other than cancer progression, the same schedule should be continued after the discontinuation of the investigational treatment.

8 DATA COLLECTION

8.1 Enrollment Number

Enrollment numbers assigned at the time of enrollment should be used for identifying the subjects.

8.2 Case Report Form (CRF)

8.2.1 Types of CRFs

1) Enrollment and baseline records
2) Treatment records
3) Laboratory tests
4) Clinical findings
5) Concomitant therapies and supportive therapies
6) Report of treatment completion
7) Imaging test records
8) Follow-up investigation

8.2.2 Completing the CRF

In this study, a system (EDC) to electronically prepare CRFs will be used, and CRFs will be electronically recorded.

Preparation of the CRF using the EDC system

1) The sponsor-investigator (investigator) and other relevant persons should use the EDC system and enter data in the CRF.
2) The investigator should ensure that CRFs to be submitted are accurate, complete and sent in a timely manner, and that enrollment numbers are utilized for identifying the subjects.
3) Data contained in the CRF, which are based on source documents, shall be consistent with the source documents. If there is any inconsistency with source documents, the investigator/subinvestigator should prepare a record describing its reason, submit it to the coordinating investigator and keep its copy. If there is any change or modification in the record on the CRF, the investigator/subinvestigator should make the change or modification in accordance with a manual. Changes or modifications shall be made in such a way to keep the originally documented information legible.

8.3 Method for the Collection of CRFs

CRFs will be collected by sending them through the EDC system. Timing for their submission will be in accordance with separately specified "EDC input guidelines."

9 SAFETY HANDLING

9.1 Definition of Adverse Event (AE)

An AE is any untoward medical occurrence during and after treatment with a drug or worsening of preexisting symptoms or signs and includes any event which does not necessarily have to have a causal relationship with this drug treatment. The term, untoward medical occurrence, refers to symptoms (e.g., nausea and chest pain), signs (e.g., tachycardia and hepatomegaly) or test result (e.g., laboratory values and ECG) abnormalities. In clinical studies, an AE is any untoward medical occurrence during the study period including an observation or washout period even if no investigational drug is administered.

9.2 Definition of Serious Adverse Event (SAE)

A serious adverse event (SAE) is that meets at least one of the following criteria at any dose of the study or control drug:

- An event that results in death;
- An event that is life-threatening;
- An event that requires inpatient hospitalization or prolongation of existing hospitalization
- An event that results in persistent or significant disability/incapacity;
- An event that is a congenital anomaly/birth defect; or
- An important medical event that may jeopardize the subject or may require medical intervention to prevent one of the other outcomes listed in the definition above.

The progression of cancer, which is the target disease of this clinical study, including its signs and symptoms will not be deemed as SAEs. It is not necessary to report hospitalization attributable to the signs and symptoms of disease progression as an SAE.

Other AEs that are determined to be medically important conditions:

Other important AEs are AEs other than SAEs and AEs leading to the discontinuation of the investigational drug in subjects, and clinically particularly important AEs are classified into “other important AEs.”

“Other important AEs” include the following AEs:

“Other important AEs” for atezolizumab

- Pneumonitis
- Colitis
- Endocrine disorders: Diabetes mellitus, pancreatitis, adrenal insufficiency, hyperthyroidism and hypophysitis

Seto T, et al. J Immunother Cancer 2022; 10:e004025. doi: 10.1136/jitc-2021-004025

- Hepatitis (including increased AST or ALT > 10 x upper limit of normal [ULN])
- Systemic lupus erythematosus
- Nervous system disorders: Guillain-Barre syndrome, myasthenic syndrome, myasthenia gravis and meningoencephalitis
- Hypersensitivity, cytokine release syndrome, infusion reaction, influenza like illness, systemic inflammatory response syndrome or events suggesting excessive systemic immune response
- Nephritis
- Ocular toxicity (e.g., uveitis and retinitis)
- Myositis
- Myopathy (including rhabdomyolysis)
- Grade ≥ 2 cardiac disorders (e.g., atrial fibrillation, myocarditis and pericarditis)
  - In the case of suspecting drug-induced liver injury such as an increase in bilirubin values as defined by the Hy's law, or increased ALT or AST with clinical jaundice.
  - Transmission of infectious pathogens that may be mediated by atezolizumab (It is defined as follows:)
  - All microorganisms, viruses or infective particles (e.g., prion protein transmitting transmissible spongiform encephalopathy) are deemed as infectious agents whether they are pathogenic or non-pathogenic. When clinical symptoms or laboratory findings suggesting infection are noted in subjects exposed to atezolizumab, the spread of infectious agent may be suspected. This term is applicable only to the case where contamination with the investigational drug is doubted.

"Other important AEs" for bevacizumab

- Digestive tract perforation including digestive tract fistula/abscess (any grade)
- Fistula/abscess other than those in the digestive tract (Grade ≥ 2)
- Hemorrhage (Grade ≥ 3) (central nervous system hemorrhage, any grade; hemoptysis, Grade ≥ 2)
- Wound healing complications (Grade ≥ 3)
- Arterial thromboembolism (any grade)
- Venous thromboembolism (Grade ≥ 3)
- Hypertension (Grade ≥ 3)
- Posterior reversible encephalopathy syndrome (PRES) (any grade)
- Proteinuria (Grade ≥ 3)
- Congestive heart failure (CHF)/ left ventricular systolic dysfunction (Grade ≥ 3)
- Bilirubin increased or increased ALT or AST with clinical jaundice (For the definition, see the Section "11.6.6. Liver function test abnormalities.")

- Transmission of infectious pathogens that may be mediated by bevacizumab (It is defined as follows:)
  - All microorganisms, viruses or infective particles (e.g., prion protein transmitting transmissible spongiform encephalopathy) are deemed as infectious agents whether they are pathogenic or non-pathogenic. When clinical symptoms or laboratory findings suggesting infection are noted in subjects exposed to bevacizumab, the spread of infectious agent may be suspected.

9.3 Evaluation of AEs

For AEs, the CTCAE version 4.0, Japanese translation version by the JCOG/JSCO will be used.

When grading AEs, a grade, which is closest to the definition of each grade, should be chosen. For the examination on AEs noted for treatment-related deaths and the causal relationship with death, an AE report should be sent, and they should be documented in the column for "Conditions at the time of death" of the Report of Treatment Completion Form and the Follow-up Investigation Form.

9.4 Assessment of the Causal Relationship

The investigator/subinvestigator shall assess the causal relationship between the investigational drug and AE and specify either "Yes" or "No" to a question: "Do you think that there is a reasonably possibility that AE is caused by the investigational drug?"

For SAEs, the causal relationship with other treatments and clinical study procedures should be assessed as well. If SAEs are likely to be related to the clinical study procedures, the causal relationship should be assessed as "related."

9.5 Actions Taken at the Onset of AEs and Follow-up Investigation

The investigator/subinvestigator should follow up subjects for AEs during the investigational treatment period and for 28 days after the last dose, and properly take actions and perform a follow-up investigation if AEs occurred.

A follow-up investigation should be carried out for any AE noted within 28 days after the last dose until its resolution except for the case where the AEs are assumed not to resolve due to underlying diseases (preexisting diseases such as complications) in the subjects on the judgement of the investigator/subinvestigator or where there are justifiable reasons such as subsequent treatment, not making visits and death. When no follow-up investigation can be implemented, such a fact should be recorded in medical charts. From 28 days after the last dose of the investigational drug and onwards, all deaths suspected to be related to the investigational drug, SAEs, and non-serious AEs of

special interest should be handled as specified in the Sections 9.6, 9.7 and 9.8.

9.6 AEs That Are Required to Be Reported (Reporting of SAEs)

During the clinical study, when learning the onset of SAEs, the investigator/subinvestigator or other clinical study collaborator shall immediately (within 24 hours) report the AEs to the coordinating investigator using a detailed report ("[Medical] Form 12-1, 2 SAE Report") in accordance with separately stipulated "Procedures for the handling of safety information" and notify them to the head of the study site as well according to the rules of the study site.

Essential information required when the investigator/subinvestigator make the initial reporting of an SAE is an enrollment number or subject ID code, AE term, seriousness and the date of onset. In addition, the following detailed information shall be reported as soon as it is obtained:

- Severity
- Outcome (date of resolution if possible)
- Causal relationship (causal relationship with the investigational drug and if applicable with concomitant medications)
- Date when the AE became serious
- Discontinuation of the investigational drug
- Action taken for the AE
- Concomitant therapies (excluding treatment for the AE)
- Concomitant medications (If the causal relationship with the AE cannot be assessed, premedications shall be included.)
- Date of birth and gender
- Current medical history (complications)
- Relevant past history
- Date of death and course until death if applicable

Two or more AEs may be recorded as AEs leading to discontinuation or death. When the cause of death is unknown, it should be handled as "death of unknown cause." If an autopsy has been carried out, a copy of its report should be, as necessary, submitted.

The coordinating investigator shall report SAEs, which have been notified by the investigator/subinvestigator or investigational drug supplier, to the investigators/ sponsor-investigators at the other study sites and the investigational drug supplier. The investigators/sponsor-investigators at the other study sites and the investigational drug supplier shall individually inform the coordinating investigator of receiving such information.

The investigator/subinvestigator shall also immediately (within 24 hours) report

Seto T, *et al. J Immunother Cancer* 2022; 10:e004025. doi: 10.1136/jitc-2021-004025

follow-up information on SAEs or events, which were non-serious AEs but subsequently found to be serious, in accordance with the above procedures.

9.7 Reporting to the Minister of Health, Labour and Welfare

When it is necessary to notify an AE learned as described in the Section "9.6 AEs That Are Required to Be Reported (Reporting of SAEs)" to the Minister of Health, Labour and Welfare, the coordinating investigator should prepare a report in accordance with the separately stipulated "Procedures for the handling of safety information" in coordination with the investigator and submit it to the Pharmaceuticals and Medical Devices Agency (PMDA) within the specified period.

9.8 Reporting to the Investigational Drug Supplier

The coordinating investigator shall report SAEs notified as set forth in the Section "9.6 AEs That Are Required to Be Reported (Reporting of SAEs)" to the investigational drug supplier within one business day after the coordinating investigator received the information. Also, if a report has been submitted to the PMDA as mentioned in the Section "9.7 Reporting to the Minister of Health, Labour and Welfare," the coordinating investigator shall hand in its copy to the investigational drug supplier.

In addition, pregnancy shall be reported to the investigational drug supplier in accordance with the Section "9.9 Actions at the Time of Pregnancy" and separately stimulated "Memorandum on the reception of safety management information." Overdosing shall be also notified to the investigational drug supplier in accordance with the separately specified "Procedures for the handling of safety information."

9.9 Actions at the Time of Pregnancy

Exposure to women

If female subjects become pregnant during the protocol treatment, the protocol treatment should be immediately discontinued.

Pregnancy will not be deemed as an AE except for the case where the effect of contraceptives is assumed to be reduced by the investigational drug. Congenital anomaly and spontaneous abortion should be reported as SAEs. Abortion without complications will not be handled as an AE. However, for the outcome (spontaneous abortion, abortion, ectopic pregnancy, normal birth or congenital anomaly) of all pregnancies, including the case where the subjects have been withdrawn from the clinical study, a follow-up investigation should be implemented, and its record should be kept. When learning pregnancy by 5 months from the last dose of atezolizumab or 6 months from the last dose of bevacizumab whichever is later, the investigator/subinvestigator should immediately (within 24 hours after identifying it)

report the event to the coordinating investigator. The coordinating investigator should, in collaboration with the investigator/subinvestigator, provide all related information on an overseas safety data entry site of the investigational drug supplier. When obtaining information on outcome, the same reporting period will be applied.

Exposure to men

Pregnancy of the partners of male subjects will not be deemed as an AE. If possible, follow-up investigations on the outcome (spontaneous abortion, abortion, ectopic pregnancy, normal birth or congenital anomaly) of all pregnancies should be carried out, and its record should be kept. When obtaining information on pregnancy from the partners of male subjects, consent for the collection of information related to pregnancy and its outcome shall be obtained from the partners of male subjects. With regard to pregnancy of the partners of subjects occurred during a period from treatment initiation to 6 months after treatment end, a follow-up investigation should be implemented for its outcome, and its record should be kept.

10 RESPONSE ASSESSMENT AND ENDPOINTS

10.1 Definitions of Endpoints

10.1.1 Overall survival (OS)

OS is time to death of any cause by defining the day of enrollment as the starting date of the computation. Subjects, who are alive at the completion of the follow-up period and who are lost to follow-up, will be censored on the day of last survival confirmed.

10.1.2 Progression free survival (PFS)

PFS is time until the day of death of any cause, the day of an imaging test revealing progression, or the day when progression is diagnosed in clinical practice, whichever is earlier by defining the day of enrollment as the starting date of the computation.

Progression is defined as clear progression as determined by imaging tests. In an investigator-assessment, obvious progression (clinical progression) based on physical findings is also deemed as progression.

Subjects, whose death or progression is not confirmed at the completion of the follow-up period and for whom the days of reaching these events are unknown, will be censored on the day of the latest outpatient visit or the day of medical examination during hospitalization before they were lost to follow-up.

Seto T, *et al. J Immunother Cancer* 2022; 10:e004025. doi: 10.1136/jitc-2021-004025

10.1.3 Objective response rate (ORR)

An antitumor activity should be assessed in accordance with the “New response evaluation criteria in solid tumors: Revised RECIST guideline (version 1.1) - Japanese translation version by the JCOG - (RECIST v1.1 Japanese translation by the JCOG).” For determining complete response (CR) and partial response (PR) as the best overall response, it is required to finalize it on the basis of the duration of response that is maintained for at least 4 weeks. For assessing the best overall response as stable disease (SD), it is required that overall response is SD during a period from enrollment to Week 6.

Response at each time point should be evaluated in accordance with Tables 1, 2 and 3 in the Section “4.4.1. Time point response” of the RECIST version 1.1 (However, no tumor marker will be used for determining CR for non-target lesions.).

The baseline evaluation should be carried out using imaging tests at baseline. An ORR refers to the proportion of subjects who achieved CR or PR in an analysis population.

Results by the central assessment will be adopted because a bias for ORR by researchers appears.

10.1.4 Duration of response (DOR)

The DOR is a period from the day when CR or PR as the best overall response is recorded for the first time to the day of an imaging test revealing progression, the day when progression is diagnosed in clinical practice, or the day of death (regardless of the cause of death) whichever is earlier.

11 STATISTICAL PROCEDURES AND ANALYSIS METHODS

Statistical analyses are summarized below. More detailed analysis methods are provided in separately specified statistical analysis plan.

11.1 Analysis Populations

Analysis populations in this clinical study are as defined below. The handling of each subject should be discussed and decided between the coordinating investigator and statistical analysis manager before data fixation.

Full analysis set (FAS): The FAS will be composed of all enrolled subjects who received at least one dose of the investigational drug and are assessed for efficacy for at least once.

Tumor response evaluable set: The tumor response evaluable set will be composed of

all enrolled subjects who received at least one dose of the investigational drug and have measurable lesions based on review by the investigator using baseline imaging data. In the tumor response evaluable set, the primary analyses of the ORR as determined by the investigator in accordance with the RECIST guideline version 1.1 and other endpoints based on the RECIST will be performed.

Safety analysis set: The safety analysis will be composed of all enrolled subjects who received at least one dose of the investigational drug.

11.2 Data Handling

11.2.1 Handling of protocol deviation data

The handling of deviations requiring discussion should be decided by the coordinating investigator.

11.2.2 Handling of missing, non-adopted and abnormal data

The tests and observations, which have never been performed, should be handled as missing data. No data will be imputed.

11.3 Summary of the Implementation Status of the Clinical Study

The enrollment status, major protocol deviations (including major deviations from the inclusion/exclusion criteria), and reasons for withdrawal from the study will be summarized. In addition, compliance with the protocol treatment and reasons for the discontinuation of the investigational treatment will be summarized for subjects who received the protocol treatment.

11.4 Baseline Characteristics

Baseline characteristics will be descriptively summarized. For continuous data, the mean, median, standard deviation, minimum and maximum will be presented. For categorical data, frequency and proportions will be provided.

11.5 Efficacy Analysis

11.5.1 Primary efficacy endpoint

In the primary efficacy analysis of this clinical study, the ORR for atezolizumab + bevacizumab therapy will be assessed. In the tumor response evaluable set, the observed ORR and its 90% CI (exact method) will be calculated. If the lower limit of the 90% CI is above the threshold ORR of 40%, the therapy will be determined to be effective. Just for reference, 95% CIs will be also calculated.

Seto T, *et al. J Immunother Cancer* 2022; 10:e004025. doi: 10.1136/jitc-2021-004025

11.5.2 Secondary efficacy endpoints

For PFS, OS and DOR, Kaplan-Meier curves will be generated, and the median and its 95% CI will be presented. Also, point estimates of PFS rates at Month 6, Year 1 and Year 2 and their 95% CIs, and point estimates of 1-year and 2-year survival rates and their 95% CIs will be estimated.

11.10 Safety Analysis

For all AEs, the incidence by grade and incidence of Grade 3 + Grade 4 events and their proportions will be determined using the CTCAE version 4.0 Japanese translation version by the JCOG/JSCO. In addition, the incidence by Preferred Term (PT) and System Organ Class (SOC) of the Medical Dictionary for Regulatory Activities (MedDRA)/J, and their proportions will be calculated.

11.11 Interim Analysis

No interim analysis will be performed. However, interim tabulations of efficacy and safety data may be carried out at the time when a certain number of subjects are collected not for the purpose of changing the study design, analysis plan or other relevant matters of this study but for the purpose of determining moving on to the next phase earlier.

12 ETHICAL MATTERS

All researchers related to this clinical study will conduct the study in accordance with the Declaration of Helsinki (amended in Fortaleza in October 2013), the standards specified in Article 14, Paragraph 3 and Article 80-2 of the Pharmaceuticals, Medical Devices, and Other Therapeutic Products Act, and the “Ministerial Ordinance on Good Clinical Practice (GCP)” (Ordinance No. 9 of the Ministry of Health, Labour and Welfare dated January 22, 2016).

Also, all the said researchers shall comply with the study protocol unless interfering with the safety or human rights of the subjects.

12.1 Protection of the Privacy of the Subjects

Information, which may identify the subjects such as the name of a subject, should not be notified from the study site to the coordinating investigator and other relevant persons.

The subjects should be identified or checked using enrollment numbers to be issued at the time of enrollment, subject ID codes*, gender and/or date of birth so that any third party can identify the subjects such as their names.

In addition, prepared CRFs and other relevant documents shall be used only for the purpose of this study.

* Subject ID code: A subject ID code refers to a number (code) used when the study site provides a subject's information to the external party.

12.2 Receiving Informed Consent

The investigator/subinvestigator should adequately explain the items listed below to the subjects using written information for subjects, which has been determined by the head of the study site based on the approval of the institutional review board (IRB), prior to their enrollment. Also, the investigator/subinvestigator should give the subjects an opportunity to ask questions and an ample of time to decide whether or not to participate in the study.

After confirming that the subjects have fully understood the contents of the study, written voluntary consent for participation in the study should be received personally from the subjects.

The investigator/subinvestigator should affix his/her seal or signature to an informed consent form and promptly hand a copy of the dated informed consent form to the subject. The original of the informed consent form should be kept in his/her medical chart.

12.3 Matters to Be Explained to Patients Using the Written Information for Subjects

(1) That the study involves research.
(2) The purpose of the study.
(3) The name, title and contact address of the investigator or subinvestigator
(4) The method of the study (including those aspects of the study that are experimental, inclusion/exclusion criteria for subjects, and the probability for random assignment to each treatment if randomization is performed).
(5) The expected clinical benefits and risks or inconveniences to the subject (When there is no intended benefit to the subject, the subject should be made aware of this.). The subject shall be notified all the latest information related to foreseeable risks based on the clinical study.
(6) If the subjects are patients, the alternative procedure(s) or course(s) of treatment that may be available to the subject, and their important potential benefits and risks.
(7) The expected duration of the subject's participation in the study.
(8) That the subject's participation in the study is voluntary and that the subject or his/her representative may refuse to participate or withdraw from the study, at

any time, without penalty or loss of benefits to which the subject is otherwise entitled.

(9) That the monitor(s), the auditor(s), the IRB, and the regulatory authority(ies) will be granted direct access to the subject's original medical charts without violating the confidentiality of the subject, and that, by signing the informed consent form, the subject or his/her representative is authorizing such access.
(10) If the results of the study are published, the subject's identity will remain confidential.
(11) The person(s) at the study site to contact for further information regarding the study and the rights of subjects, and whom to make inquiries or contact in the event of study-related injury.
(12) The compensation and/or treatment available to the subject in the event of study-related injury.
(13) Type of the IRB investigating and reviewing the appropriateness of the study, matters to be reviewed by each IRB, and other study-related matters concerning the IRB.
(14) That the subject may check written procedures and other relevant documents of the IRB and should make a request if he/she wishes to do so. If the written procedures, etc. of the IRB are disclosed on a website, its address, and if they are not disclosed, the written procedures, etc. are accessible to the public.
(15) The approximate number of subjects involved in the study.
(16) That the subject or his/her representative will be informed immediately if information becomes available that may be relevant to the subject's or his/her representative's willingness to continue participation in the study.
(17) The foreseeable circumstances and/or reasons under which the subject's participation in the study may be terminated.
(18) The anticipated expenses, if any, to the subject for participating in the study.
(19) The anticipated payment (e.g., agreement on calculating the amount of payment), if any, to the subject for participating in the study.
(20) The subject's responsibilities
(21) Matters concerning intellectual property rights
(22) Matters concerning the conflict of interest (COI)
(23) Other necessary matters

12.4 Preparation and Revision of the Written Information for Subjects and Informed Consent Form/ Provision of Information to the Subjects

1) The investigator at each study site should prepare the site version of written information for subjects and an informed consent form using the samples of the written information for subjects and an informed consent form created by the

coordinating investigator.

2) If information, which may affect the subjects' willingness to continue taking part in the study, is obtained, the investigator or subinvestigator should promptly notify the information to the subjects and confirm their willingness to continue participating in the study. Also, the investigator or subinvestigator should record the date of providing the information to the subject, the details of the notified information and the confirmation result in the original medical records such as a medical chart.
3) When it is found to be necessary to amend the informed consent form and written information for subjects (if new important information, which may be relevant to the subjects' consent, is obtained), the investigator should promptly amend the informed consent form and written information for subjects based on the concerned information and receive the approval of the IRB beforehand. In addition, the investigator and subinvestigator should give an explanation again to all subjects participating in the study using the revised written information for subjects and receive written voluntary informed consent for continue taking part in the study from the subjects.

12.5 Approval of the IRB

When participating in the clinical study, a decision of the head of the study site based on the approval of the IRB at each study site for the appropriateness of conducting the study shall be obtained.

When approval is obtained, the original of an approval certificate should be properly retained at the study site, its copy should be sent to the coordinating investigator, and the coordinating investigator should properly keep the copy.

12.6 Review on Continuation by the IRB

1) The investigator should submit a written summary of the current status of the clinical study once a year or more frequently upon request of the IRB to the head of the study site to undergo a review on continuation by the IRB.
2) The head of the study site should seek opinion on the continuation of the clinical study from the IRB in the following cases where: A report on the current status of the study or reports on adverse drug reactions (ADRs) during the study are received; deaths, which may be attributable to ADRs associated with the investigational drug, or other SAEs occurred and reported by the investigator; information, which may affect the subjects' willingness as to whether or not to remain participating in the study, is obtained; and such is found to be necessary for other reasons.
3) When a monitoring or audit report for the study is received, the head of the

study site should seek the opinion from the IRB on the appropriateness of conducting the study at the concerned study site.

12.7 Responsibilities and Compensation for Injuries to the Subjects

In the case where any injury attributable to the conduct of the study occurred in the subjects, the investigator/sponsor-investigator should give the best treatment to the subjects. When it is determined to be an unknown SAE attributable to the study, it should be handled in accordance with a separately specified summary of the compensation system. Furthermore, the subjects' health insurance will apply to other necessary measures for injuries.

13 MONITORING AND AUDITS

13.1 Monitoring

The sponsor-investigator should prepare written monitoring procedures and have monitors perform monitoring in accordance with the written procedures while taking account of the opinions of the IRB at the study site to verify that the protection of human rights, the maintenance of safety, and the improvement of welfare for the subjects are implemented, that the study has been conducted in compliance with the latest protocol and GCP Ordinance, and that it can be validated that clinical study data and other relevant items reported by the investigator or subinvestigator are accurate and complete as compared with study-related records such as source documents.

13.2 Monitoring Reports

1) When it is identified that the study at the study site is not carried out in accordance with the GCP Ordinance or protocol as a result of monitoring, a monitor should immediately notify such a fact to the investigator at the study site.
2) When on-site monitoring was performed, monitors should submit a monitoring report presenting matters listed below at each time to the sponsor-investigator, the head of the study site pertaining to the monitoring, and the coordinating investigator:
   (1) Date and time when the monitoring was implemented;
   (2) Names of monitors;
   (3) Name of the investigator/subinvestigator to whom an explanation was asked at the time of monitoring;
   (4) Summary of monitoring results;
   (5) Matters informed to the investigator pursuant to the provisions of the preceding paragraph; and
   (6) Measures to be taken for the matters set forth in the preceding item and

Seto T, *et al. J Immunother Cancer* 2022; 10:e004025. doi: 10.1136/jitc-2021-004025

monitoring findings on the said measures

The status of the progress of the study, enrollment eligibility, safety and other relevant matters should be reported to the sponsor-investigator and the head of the study site pertaining to monitoring.

13.3 Audits

The sponsor-investigator should prepare a written plan and operating procedures for audits to ensure the quality of the study and evaluate whether or not the study being conducted in compliance with the GCP Ordinance, protocol and procedures independently and separately from usual monitoring and quality control activities for the clinical study, and have auditors perform audits in accordance with the plan and procedures while taking account of the opinions of the IRB at the study site.

13.4 Audit Reports

When an audit was carried out, auditors prepare an audit report recording matters verified at the audit and audit certificate demonstrating that the audit has been conducted, and submit them to the sponsor-investigator, the head of the study site, and the coordinating investigator.

14 QUALITY CONTROL AND ASSURANCE FOR THE STUDY

14.1 Data Quality Control

The sponsor-investigator should implement the quality control of this study, and keep and retain control records to ensure the implementation and safety of the study, and the accuracy and reliability of data.

14.2 Data Quality Assurance

The quality assurance of the study should be ensured by auditors in accordance with the Sections “13.3. Audits” and “13.4. Audit Reports.”

14.3 Access to Records

The head of the study site shall collaborate in monitoring and audits implemented by the sponsor-investigator and inspections by the IRB and other relevant parties. When monitoring, audits or inspections are to be carried out, the head of the study site should grant access to all study-related records such as source documents upon request of the monitors, auditors, IRB and other relevant parties.

14.4 Data Handling and Retention of Records

14.4.1 Handling of the CRF and data

The study site should pay careful attention to the protection of personal information with regard to the handling of CRFs or test reports and their copies to prevent the leakage, loss, transcription and unauthorized copying of the information.

14.4.2 Retention of records

CRFs or test reports and their copies should be archived until the days stipulated below.

1) Study site
Essential documents, which should be retained by the head of the study site or the IRB, should be properly kept by a record archiving manager designated by the head of the study. The duration of archiving shall be the day specified in the following Item (1) or (2) whichever is later. When the sponsor-investigator needs to retain these documents at the study site for a period longer than this, the actions for the archiving period and method should be discussed with the sponsor-investigator.
    (1) Date of marketing approval for the investigational drug (when development discontinuation or the fact of not attaching the results of the clinical study to an approval application is notified, the day 3 years from the day of receiving such a notification)
    (2) Day 3 years after the discontinuation or completion of the study

2) Sponsor-investigator
The sponsor-investigator should properly retain study-related records for a period until the day set forth in the following Item (1) or (2) whichever is later in accordance with separately stipulated "Procedures for record archiving." The sponsor-investigator may request the head of the study site, with which he/she affiliates, to carry out activities for retaining the records. When the sponsor-investigator is disengaged from his/her affiliated study site, the head of his/her affiliated study site may take charge of the record archiving activities.
When it is no longer necessary to archive the records, the sponsor-investigator shall notify such a fact to the head of the study site or the IRB organizer via the head of the study site.
    (1) The day when the investigational drug supplier receives drug marketing approval for the investigational drug (when development discontinuation or the fact of not attaching the results of the clinical study to an approval application is notified, the day 3 years from the day of receiving such a

notification)

(2) Day 3 years after the discontinuation or completion of the study

In the case where drug approval is granted for the investigational drug, the sponsor-investigator should take necessary measures for the handling of the said records such as concluding a contract with the investigational drug supplier because an approval holder is required to archive the said records for a given period pursuant to the provisions of Article 101 of the Enforcement Regulations of the Pharmaceuticals, Medical Devices, and Other Therapeutic Products Act.

3) Investigator
The investigator should retain study-related documents as instructed by the head of the study site.

15 CHANGES, DISCONTINUATION AND TERMINATION CONCERNING THE CONDUCT OF THE STUDY

15.1 Protocol Revision

1) When it is found to be necessary to amend the protocol other than administrative matters (e.g., modifications in texts such as a change of a telephone number) of the study, the sponsor-investigator should make amendments after discussing the appropriateness of the changes and their effects on study evaluation with the other sponsor-investigators and coordinating investigator, and, as necessary, efficacy and safety evaluation committee and other relevant parties.
2) The sponsor-investigator should promptly notify the details of protocol amendments to all the heads of the study sites and the coordinating investigator, and take procedures specified at each study site.

15.2 Protocol Deviations

1) The investigator or subinvestigator at each study site shall not make any protocol deviation or change before the investigator obtains written the approval of the IRB based on its prior review. However, this is not applicable to the case where it is necessary to make a deviation or change due to inevitable medical reasons such as avoiding immediate hazard to the subjects.
2) The investigator or subinvestigator should record all deviations from the protocol regardless of their reasons.
3) The investigator or subinvestigator may implement a protocol deviation or change when such is necessary for inevitable medical reasons such as avoiding immediate hazard to the subjects. In such a case, the investigator should immediately submit a document presenting such a fact and the reason to the head

of the study site to promptly report such a fact and the reason to the IRB and other relevant parties via the head of the study site.

15.3 Premature Termination and Suspension of the Conduct of the Study

When prematurely terminating or suspending the study, the sponsor-investigator should discuss with the coordinating investigator, decide it, and promptly report in writing such a fact and the reason to the head of the study site and the regulatory authority.

The term, premature termination, refers to the discontinuation of all or part of the study earlier than planned due to any of the following reasons:

- When the discontinuation of the study is determined;
- When it is judged that there are safety concerns for the study;
- When the significance of the study is defined; or
- When it is determined to be difficult to complete the study due to a delay in subject enrollment.

The follow-up period in the case of premature termination will be as described in the protocol by defining the last day of enrollment as the starting date of the computation.

Even if the study is prematurely terminated, the sponsor-investigator should discuss with the investigational drug supplier that the subjects can continue the investigational treatment if requested by the subjects unless it interferes with treatment.

15.4 Premature Termination and Suspension at the Study Site

When the study site is found to have interfered with the proper conduct of the study by violating the GCP Ordinance and protocol (excluding the case where it is necessary to avoid immediate hazards to the subjects and due to other inevitable medical reasons), the sponsor-investigator should prematurely terminate the study at the study site by giving a prior notice on such to the coordinating investigator. When suspending or prematurely terminating the study, the sponsor-investigator should promptly report in writing such a fact and the reason to the head of the study site and the coordinating investigator. The coordinating investigator should promptly inform such a fact and the reason to the sponsor-investigators at the other study sites. When the study was prematurely terminated due to incompliance, the sponsor-investigator should promptly notify it to the regulatory authorities.

When the sponsor-investigator reports that the study has been suspended or will be prematurely terminated, the head of the study site should promptly notify it in writing to the IRB and provide a detailed explanation.

15.5 Efficacy and Safety Evaluation Committee

The efficacy and safety evaluation committee will be established for objectively evaluating the efficacy and safety in the study, and suggesting the continuation, changes in or premature termination of the study to the sponsor-investigator from the ethical and scientific perspectives. The efficacy and safety evaluation committee should be operated in accordance with separately specified “Procedures for the efficacy and safety evaluation committee.”

16 STUDY END AND ITS REPORTING

After the end of the study, the investigator should inform in writing the head of the study site that the study has been completed and report a summary of study results in writing. When the investigator has reported that the study will be completed, the head of the study site should notify in writing such a fact and a summary of its results to the IRB.

17 BURDEN OF STUDY EXPENSES

17.1 Study Operation Cost

Chugai Pharmaceutical Co., Ltd. will support expenses required for operating this clinical study and supply investigational drugs.

17.2 Expenses Required for the Protocol Treatment

The health insurance of the subjects will be applied to expenses for tests and diagnostic imaging during the study period and expenses for concomitant medications used for the study. Also, the health insurance of the subjects will be applied to other necessary measures for health injuries.

18 MATTERS CONCERNING THE CONFLICT OF INTEREST (COI)

This study will be aided by Chugai Pharmaceutical Co., Ltd. but implemented as an investigator-initiated clinical study. The COI for researchers who are involved in the study and persons who are supporting West Japan Oncology Group (WJOG) clinical studies should be managed as follows:

1) The COI for persons relevant to the study should be controlled as stipulated at the participating sites.
2) The COI for persons who serve the central roles of the study such as the coordinating investigator, Group leader and president should be managed by the WJOG ethics committee.

3) The COI for WJOG secretariat staffs other than the above should be controlled in a similar manner.

19 PUBLICATION OF STUDY RESULTS AND ATTRIBUTION OF RESULTS

19.1 Publication of Results

After study completion, its results will be summarized, adjusted by the coordinating investigator and sponsor-investigator/investigator, and then presented at appropriate Japanese and/or overseas academic conferences and published in scientific journals.

19.2 Clinical Study Report

After study completion, the coordinating investigator committee and sponsor-investigator will adjust and prepare a clinical study report in accordance with separately specified "Procedures for the preparation of a clinical study report."

19.3 Intellectual Property Rights

The designs of the protocol, enrollment form and CRF, database files created as a result of executing the study and forms obtained from them will belong to the WJOG. The intellectual property rights for the invention of the investigational drug* belongs to Chugai Pharmaceutical Co., Ltd.

Intellectual property arising from an exploratory study will belong to the concerned researchers.

In the case where intellectual property including patent rights (excluding the intellectual property rights for the invention of the investigational drug) arises from the implementation of the study, it will be divided between the WJOG and study sites according to the degree of their contribution.

* The term "invention of the investigational drug," refers to all inventions concerning the investigational drug (including the novel indications or dosage regimens of the investigational drug but not limited to these) that have been contrived or created by the WJOG, investigator or study site-related persons individually or jointly with other persons, or made using other methods with respect to the investigator-initiated clinical study. The invention of the investigational drug includes invention related to (a) the metabolic activity, pharmacological activity, ADRs, drug metabolism, mechanism of action, safety and drug interactions for the investigational drug, or (b) biomarkers, tests, diagnostic methods or diagnostics used for predicting subjects' response or tolerability to the investigational drug or utilized using methods to select subjects to be treated with the investigational drug. However, intellectual properties obtained

through accessory studies will belong to the researchers of the accessory studies.

19.4 Secondary Use of Data

Data, excluding personal information, may be secondarily used if the coordinating investigator, sponsor-investigator or WJOG board of (permanent) directors judges that it is beneficial to secondarily use data obtained from the study for statistical analyses, meta-analyses, etc.

19.5 Provision of Data

Prior to the end of the study, interim tabulations of efficacy and safety data, which are carried out at the time when a certain number of subjects are collected not for the purpose of changing the study design, analysis plan or other relevant matters of this study but for the purpose of determining moving on to the next phase earlier, may be provided to the funding company. After the end of the study, anonymized study data and forms may be provided with or without charge upon instruction of the regulatory authorities or request of relevant companies.

20 PRIOR REGISTRATION OF THE STUDY PLAN

The WJOG will register this study to the University Hospital Medical Information Network-Clinical Trials Registry (UMIN-CTR) system prior to its implementation.

21 STUDY IMPLEMENTATION STRUCTURE

See a separately volume “Study implementation structure.”

22 OTHERS

1) Training to study staffs
   The sponsor-investigator/investigator should give training on procedures for the study and a system to be used to staffs related to the study at the study site before enrolling the first subject at the study site, and record its results.
2) Contract between the WJOG and sites
   The WJOG should conclude a support agreement with each study site for the purposes of supporting the conduct of the study, expenses and for obtaining the investigational drug, purchasing study liability insurance, and supporting other activities entrusted from the sponsor-investigator/investigator to the coordinating investigator.

23 REFERENCES

1) Vital statistics of Japan (edited by the Statistics and Information Department,

Minister’s Secretariat, Ministry of Health, Labour and Welfare) 2016.

2) Reck M, Rodríguez-Abreu D, Robinson AG, et al. Pembrolizumab versus Chemotherapy for PD-L1-Positive Non-Small-Cell Lung Cancer. N Engl J Med. 2016 Nov 10; 375 (19): 1823-1833.
3) Shirish Gadgeel, Marcin Kowanetz, Wei Zou, et al. CLINICAL EFFICACY OF ATEZOLIZUMAB IN PD-L1 SELECTED SUBGROUPS DEFINED BY SP142 AND 22C3 IHC ASSAYS IN 2L+ NSCLC: RESULTS FROM THE RANDOMIZED OAK TRIAL. ESMO 2017.
4) Herbst RS, Baas P, Kim DW,et al. Pembrolizumab versus docetaxel for previously treated, PD-L1-positive, advanced non-small-cell lung cancer (KEYNOTE-010): a randomised controlled trial. Lancet. 2016; 387 (10027): 1540-50.
5) Rittmeyer A, Barlesi F, Waterkamp D, et al. Atezolizumab versus docetaxel in patients with previously treated non-small-cell lung cancer (OAK): a phase 3, open-label, multicentre randomised controlled trial. Lancet. 2017; 389 (10066): 255-265.
6) Borghaei H, Paz-Ares L, Horn L, et al. Nivolumab versus Docetaxel in Advanced Nonsquamous Non-Small-Cell Lung Cancer. N Engl J Med. 2015; 373 (17): 1627-39.
7) Brahmer J, Reckamp KL, Baas P, et al. Nivolumab versus Docetaxel in Advanced Squamous-Cell Non-Small-Cell Lung Cancer. N Engl J Med. 2015; 373 (2): 123-35.
8) Sandler A, Gray R, Perry MC, et al. Paclitaxel-carboplatin alone or with bevacizumab for non-small-cell lung cancer. N Engl J Med. 2006; 355 (24): 2542-50.
9) Reck M, von Pawel J, Zatloukal P, et al. Phase III trial of cisplatin plus gemcitabine with either placebo or bevacizumab as first-line therapy for nonsquamous non-small-cell lung cancer: AVAil. J Clin Oncol. 2009; 27 (8): 1227-34.
10)

24 PROTOCOL REVISION HISTORY

MM DD, 2018 Ver1.0 (approved by the WJOG board of permanent directors)

Guidelines for Risks and Adverse Events Associated with Atezolizumab

Toxicities, which are related or suspected to be related to atezolizumab treatment, should be managed in accordance with standard medical procedures. Also, additional tests such as an autoimmune antibody test and biopsy should be performed to reveal the possible involvement of the causes of immunogenicity.

The majority of immune-mediated adverse events (AEs) observed in patients treated with immune-regulating drugs are mild and spontaneously cure; however, in order to avoid the onset of significant complications, such events should be identified as early as possible and promptly treated if found. Even if atezolizumab treatment is discontinued, it does not mean that therapeutic efficacy is immediately exerted, and for severe immune-related toxicities, the management of the acute stage with external or systemic steroid treatment and/or administration of other immunosuppressive drugs may be required.

If atezolizumab treatment is to be continued, the investigator should examine a benefit-risk balance for the subject beforehand. Even for subjects who meet criteria for permanent treatment discontinuation, if there is any benefit, the reintroduction of atezolizumab may be considered after immune-mediated events completely resolved. Atezolizumab treatment may be resumed only after its approval by both investigator (or an appropriate designated person) and medical monitor is documented.

Dose modification

The atezolizumab dose will not be adjusted in this study.

Treatment interruption

In subjects who developed toxicities that are likely to be related to the protocol treatment, the protocol treatment may be interrupted. If an adrenal cortical steroid is used for the treatment of AEs, its dose shall be tapered to an oral prednisone dose of 10 mg/day or a dose less than that equivalent to this over one month or more before resuming atezolizumab. When atezolizumab treatment is interrupted for more than 105 days after the day of the onset of AE, atezolizumab should be discontinued in the subject. However, atezolizumab treatment may be interrupted for more than 105 days for tapering adrenal cortical steroids prior to resuming the treatment. Even if atezolizumab treatment is interrupted for more than 105 days, if a medical monitor judges that it may be clinically beneficial for the subjects, atezolizumab treatment may be resumed. Atezolizumab treatment may be interrupted for reasons (e.g., surgical treatment) other than toxicity, but approval of a medical monitor will be required. The acceptable duration of treatment interruption will be decided by the investigator, subinvestigator and medical monitors.

Guidelines for actions

Pulmonary events

Dyspnea, cough, fatigue, hypoxia, pneumonitis and lung infiltration have occurred in association with atezolizumab treatment. Lung-related signs and symptoms should be assessed in the subjects throughout the study period. Furthermore, chest computed tomography (CT) scans should be carried out at each time when evaluating tumors.

All pulmonary events should be adequately assessed for generally reported other causes (e.g., pneumonia/infection, lymphangiosis carcinomatosa, pulmonary embolism, heart failure, chronic obstructive pulmonary disease, and pulmonary hypertension) as well. Guidelines for the management of pulmonary events are presented in Table 1.

Table 1 Guidelines for the management of pulmonary events including pneumonitis

Event	Management
Pulmonary events, Grade 1	• Continue atezolizumab treatment, and perform thorough monitoring.<br>• Carry out an imaging test again.<br>• Consider referring the subject to a respiratory specialist.
Pulmonary events, Grade 2	• Interrupt atezolizumab treatment for 12 weeks at a maximum after the onset of event[a].<br>• Refer the subject to a respiratory specialist and infection specialist, and consider bronchoscopy or BAL.<br>• Start treatment with oral prednisone 1 to 2 mg/kg/day or at an equivalent dose.<br>• Resume atezolizumab treatment if the event improved to Grade 1 or better[b].<br>• If the event does not improve to Grade 1 or better during atezolizumab treatment interruption, permanently discontinue atezolizumab treatment, and notify it to the medical monitors[c].<br>• If the event is recurrence, handle it as a Grade 3 to 4 AE.
Pulmonary events, Grade 3 or 4	• Permanently discontinue atezolizumab treatment, and notify it to the medical monitors.<br>• Bronchoscopy or BAL is recommended.<br>• Start treatment with oral prednisone 1 to 2 mg/kg/day or at an equivalent dose.<br>• If the event does not improve within 48 hours after the start of steroid therapy, consider adding an immunosuppressive drug.<br>• If the event improved to Grade 1 or better, taper the steroid dose over one month.

BAL = bronchoalveolar lavage

[a] When it is necessary to reduce the steroid (if started) dose to an oral prednisone dose of 10 mg/day or dose less than that equivalent to this, the period of atezolizumab interruption may be extended (may be more than 12 weeks after the onset of event). The acceptable duration of prolongation shall be decided upon agreement between the investigator and medical monitor.

[b] If steroid has been started, its dose shall be tapered to an oral prednisone dose of 10 mg/day or a dose less than that equivalent to this over one month or more before resuming atezolizumab.

[c] If atezolizumab has been beneficial to subjects, the reintroduction of atezolizumab may be considered after immune-mediated events completely resolved. Atezolizumab treatment may be resumed only after its approval by both investigator (or an appropriate designated person) and medical monitor is documented.

Seto T, *et al. J Immunother Cancer* 2022; 10:e004025. doi: 10.1136/jitc-2021-004025

Hepatic events

Immune-mediated hepatitis has occurred in association with atezolizumab treatment. Patients who are eligible for the study shall have adequate liver function based on total bilirubin and observed hepatic transaminase levels. Liver function should be monitored throughout the investigational treatment period. Guidelines for the management of hepatic events are presented in Table 2.

A liver function test (LFT) should be immediately carried out in subjects who developed right upper abdominal pain and/or nausea or vomiting of unknown cause, and its results should be checked before the next dosing of the investigational drug.

For subjects with increased LFT values, their causes such as concomitant medications, viral hepatitis, and toxicity or neoplasms should be examined, and necessary actions should be taken as needed.

Table 2 Guidelines for the management of hepatic events

Event	Management
Hepatic events, Grade 1	• Continue atezolizumab treatment.<br>• Monitor LFT values until they return to normal ranges.
Hepatic events, Grade 2	**Any event:**<br>• Monitor LFT values more frequently until they return to baseline values.<br>Events persisting for more than 5 days:<br>• Interrupt atezolizumab treatment for 12 weeks at a maximum after the onset of event[a].<br>• Start treatment with oral prednisone 1 to 2 mg/kg/day or at an equivalent dose.<br>• Resume atezolizumab treatment if the event improved to Grade 1 or better[b].<br>• If the event does not improve to Grade 1 or better during atezolizumab treatment interruption, permanently discontinue atezolizumab treatment, and notify it to the medical monitors[c].
Hepatic events, Grade 3 or 4	• Permanently discontinue atezolizumab treatment, and notify it to the medical monitors[c].<br>• Consider referring the subject to a gastrointestinal specialist for undergoing a liver biopsy and assessment to identify the cause of liver injury.<br>• Start treatment with oral prednisone 1 to 2 mg/kg/day or at an equivalent dose.<br>• If the event does not improve within 48 hours after the start of steroid therapy, consider adding an immunosuppressive drug.<br>• If the event improved to Grade 1 or better, taper the steroid dose over one month.

LFT = liver function test

[a] When it is necessary to reduce the steroid (if started) dose to an oral prednisone dose of 10 mg/day or dose less than that equivalent to this, the period of atezolizumab interruption may be extended (may be more than 12 weeks after the onset of event). The acceptable duration of prolongation shall be decided upon agreement between the investigator and medical monitor.

[b] If steroid has been started, its dose shall be tapered to an oral prednisone dose of 10 mg/day or a dose less than that equivalent to this over one month or more before resuming atezolizumab.

[c] If atezolizumab has been beneficial to subjects, the reintroduction of atezolizumab may be considered after immune-mediated events completely resolved. Atezolizumab treatment may be resumed only after its approval by both investigator (or an appropriate designated person) and medical monitor is

documented.

Digestive system events

Immune-mediated colitis has occurred in association with atezolizumab treatment. Guidelines for the management of diarrhea or colitis are presented in Table 3.

All diarrhea or colitis should be fully evaluated including other more general causes. Sigmoid colonoscopy (or colonoscopy if appropriate) and a large intestine biopsy for making a definitive diagnosis of colitis and examining lymphocyte infiltration (3 to 5 samples for standard paraffin blocks) should be carried out when events persist for a long time or are severe, or when signs of systemic inflammation or acute phase reactants (e.g., increased C-reactive protein or platelet count and bandaemia) are present.

Table 3 Guidelines for the management of digestive system events (diarrhea or colitis)

Event	Management
Diarrhea or colitis, Grade 1	• Continue atezolizumab treatment.<br>• Start symptomatic treatment.<br>• Endoscopy is recommended if the symptom persists for more than 7 days.<br>• Perform thorough monitoring.
Diarrhea or colitis, Grade 2	• Interrupt atezolizumab treatment for 12 weeks at a maximum after the onset of event[a].<br>• Start symptomatic treatment.<br>• It is recommended to refer the subject to a gastrointestinal specialist.<br>• If the event has recurred or persists for more than 5 days, start treatment with oral prednisone 1 to 2 mg/kg/day or at an equivalent dose.<br>• Resume atezolizumab treatment if the event improved to Grade 1 or better[b].<br>• If the event does not improve to Grade 1 or better during atezolizumab treatment interruption, permanently discontinue atezolizumab treatment, and notify it to the medical monitors[c].
Diarrhea or colitis, Grade 3	• Interrupt atezolizumab treatment for 12 weeks at a maximum after the onset of event[a].<br>• Refer the subject to a gastrointestinal specialist for an assessment and definitive biopsy.<br>• Start treatment with methylprednisolone for intravenous injection at a dose of 1 to 2 mg/kg/day or at an equivalent dose, and switch it to oral prednisone 1 to 2 mg/kg/day or at an equivalent dose if the event improved.<br>• Resume atezolizumab treatment if the event improved to Grade 1 or better[b].<br>• If the event does not improve to Grade 1 or better during atezolizumab treatment interruption, permanently discontinue atezolizumab treatment, and notify it to the medical monitors[c].
Diarrhea or colitis, Grade 4	• Permanently discontinue atezolizumab treatment, and notify it to the medical monitors.<br>• Refer the subject to a gastrointestinal specialist for an assessment and definitive biopsy.<br>• Start treatment with methylprednisolone for intravenous injection at a dose of 1 to 2 mg/kg/day or at an equivalent dose, and switch it to oral prednisone 1 to 2

Event	Management
	mg/kg/day or at an equivalent dose if the event improved.<br>• If the event does not improve within 48 hours after the start of steroid therapy, consider adding an immunosuppressive drug.<br>• If the event improved to Grade 1 or better, taper the steroid dose over one month.

GI = gastrointestinal

[a] When it is necessary to reduce the steroid (if started) dose to an oral prednisone dose of 10 mg/day or dose less than that equivalent to this, the period of atezolizumab interruption may be extended (may be more than 12 weeks after the onset of event). The acceptable duration of prolongation shall be decided upon agreement between the investigator and medical monitor.

[b] If steroid has been started, its dose shall be tapered to an oral prednisone dose of 10 mg/day or a dose less than that equivalent to this over one month or more before resuming atezolizumab.

[c] If atezolizumab has been beneficial to subjects, the reintroduction of atezolizumab may be considered after immune-mediated events completely resolved. Atezolizumab treatment may be resumed only after its approval by both investigator (or an appropriate designated person) and medical monitor is documented.

Endocrine events

Thyroid disorder, adrenal insufficiency, diabetes mellitus and pituitary disorder have occurred in association with atezolizumab treatment. Guidelines for the management of endocrine events are presented in Table 4.

Tests to examine the presence or absence of thyroid, pituitary or adrenal endocrine disorders should be implemented in subjects who developed symptoms of unknown cause such as headache, fatigue, myalgia, impotence, constipation, and mental status changes. The subjects should be referred to an endocrinologist if endocrine disorder is suspected. Thyroid-stimulating hormone (TSH), free triiodothyronine (FT3) and free thyroxine (FT4) levels should be measured to verify the presence or absence of thyroid abnormalities. Testing of pituitary hormone levels and function (e.g., each of TSH, growth hormone, luteinizing hormone, follicle stimulation hormone, testosterone, prolactin and adrenocorticotropic hormone [ACTH] values, and ACTH stimulation test) and brain magnetic resonance imaging (MRI; detailed scanning of pituitary sections) may be helpful for differentiating between primary pituitary insufficiency and primary adrenal insufficiency.

Table 4 Guidelines for the management of endocrine events

Event	Management
Asymptomatic hypothyroidism	• Continue atezolizumab treatment.<br>• Start thyroid hormone replacement therapy.<br>• Monitor TSH every week.
Symptomatic hypothyroidism	• Interrupt atezolizumab treatment.<br>• Start thyroid hormone replacement therapy.<br>• Monitor TSH every week.<br>• Refer the subject to an endocrinologist.<br>• Resume atezolizumab treatment if the symptoms are controllable and thyroid function has improved.
Asymptomatic hyperthyroidism	In the case of TSH ≥ 0.1 mU/L and < 0.5 mU/L:<br>• Continue atezolizumab treatment.<br>• Monitor TSH every 4 weeks.<br>In the case of TSH < 0.1 mU/L:<br>• Follow the guidelines for symptomatic hyperthyroidism.
Symptomatic hyperthyroidism	• Interrupt atezolizumab treatment.<br>• As necessary, start treatment with an antithyroid drug (e.g., methimazole and carbimazole).<br>• Refer the subject to an endocrinologist.<br>• Resume atezolizumab treatment if the symptoms are controllable and thyroid function has improved.<br>• For life-threatening immune-mediated hyperthyroidism, permanently discontinue atezolizumab treatment, and notify it to the medical monitors[c].
Symptomatic adrenal insufficiency, Grades 2 to 4	• Interrupt atezolizumab treatment for 12 weeks at a maximum after the onset of event[a].<br>• Refer the subject to an endocrinologist.<br>• Perform an appropriate imaging test.<br>• Start treatment with methylprednisolone for intravenous injection at a dose of 1 to 2 mg/kg/day or at an equivalent dose, and switch it to oral prednisone 1 to 2 mg/kg/day or at an equivalent dose if the event improved.<br>• Resume atezolizumab treatment if the event improved to Grade 1 or better, and the condition became stable after F replacement therapy[b].<br>• If the event does not improve to Grade 1 or better during atezolizumab treatment interruption, or the condition does not become stable after replacement therapy, permanently discontinue atezolizumab treatment, and notify it to the medical monitors[c].
Hyperglycemia, Grade 1 or 2	• Continue atezolizumab treatment.<br>• As necessary, start insulin therapy.<br>• Monitor glucose regulation.
Hyperglycemia, Grade 3 or 4	• Interrupt atezolizumab treatment.<br>• Start insulin therapy.<br>• Monitor glucose regulation.<br>• Resume atezolizumab treatment if the symptom resolves and blood glucose

Event	Management
	levels become stable.
Hypophysitis (panhypopituitarism), Grade 2 or 3	• Interrupt atezolizumab treatment for 12 weeks at a maximum after the onset of event[a].<br>• Refer the subject to an endocrinologist.<br>• Perform brain MRI (pituitary protocol).<br>• Start treatment with methylprednisolone for intravenous injection at a dose of 1 to 2 mg/kg/day or at an equivalent dose, and switch it to oral prednisone 1 to 2 mg/kg/day or at an equivalent dose if the event improved[a].<br>• Start hormone replacement therapy according to clinical necessity.<br>• Resume atezolizumab treatment if the event improved to Grade 1 or better[b].<br>• If the event does not improve to Grade 1 or better during atezolizumab treatment interruption, permanently discontinue atezolizumab treatment, and notify it to the medical monitors[c].<br>• If hypophysitis recurred, handle it as a Grade 4 AE.
Hypophysitis (panhypopituitarism), Grade 4	• Permanently discontinue atezolizumab treatment, and notify it to the medical monitors[c].<br>• Refer the subject to an endocrinologist.<br>• Perform brain MRI (pituitary protocol).<br>• Start treatment with methylprednisolone for intravenous injection at a dose of 1 to 2 mg/kg/day or at an equivalent dose, and switch it to oral prednisone 1 to 2 mg/kg/day or at an equivalent dose if the event improved[a].<br>• Start hormone replacement therapy according to clinical necessity.

MRI = magnetic resonance imaging; TSH = thyroid-stimulating hormone

[a] When it is necessary to reduce the steroid (if started) dose to an oral prednisone dose of 10 mg/day or dose less than that equivalent to this, the period of atezolizumab interruption may be extended (may be more than 12 weeks after the onset of event). The acceptable duration of prolongation shall be decided upon agreement between the investigator and medical monitor.

[b] If steroid has been started, its dose shall be tapered to an oral prednisone dose of 10 mg/day or a dose less than that equivalent to this over one month or more before resuming atezolizumab.

[c] If atezolizumab has been beneficial to subjects, the reintroduction of atezolizumab may be considered after immune-mediated events completely resolved. Atezolizumab treatment may be resumed only after its approval by both investigator (or an appropriate designated person) and medical monitor is documented.

Seto T, *et al. J Immunother Cancer* 2022; 10:e004025. doi: 10.1136/jitc-2021-004025

Ocular events

Optical complaints (e.g., uveitis and retinal events) should be evaluated by ophthalmologists. Guidelines for the management of ocular events are presented in Table 5.

Table 5 Guidelines for the management of ocular events

Event	Management
Ocular events, Grade 1	• Continue atezolizumab treatment.<br>• It is highly recommended to refer the subjects to an ophthalmologist.<br>• Start treatment with steroid ophthalmic solutions and immunosuppressive ophthalmic solutions.<br>• If the symptom persists, handle it as a Grade 2 AE.
Ocular events, Grade 2	• Interrupt atezolizumab treatment for 12 weeks at a maximum after the onset of event[a].<br>• It is highly recommended to refer the subjects to an ophthalmologist.<br>• Start treatment with steroid ophthalmic solutions and immunosuppressive ophthalmic solutions.<br>• Resume atezolizumab treatment if the event improved to Grade 1 or better[b].<br>• If the event does not improve to Grade 1 or better during atezolizumab treatment interruption, permanently discontinue atezolizumab treatment, and notify it to the medical monitors[c].
Ocular events, Grade 3 or 4	• Permanently discontinue atezolizumab treatment, and notify it to the medical monitors.<br>• Refer the subjects to an ophthalmologist.<br>• Start treatment with oral prednisone 1 to 2 mg/kg/day or at an equivalent dose.<br>• If the event improved to Grade 1 or better, taper the steroid dose over one month.

a When it is necessary to reduce the steroid (if started) dose to an oral prednisone dose of 10 mg/day or dose less than that equivalent to this, the period of atezolizumab interruption may be extended (may be more than 12 weeks after the onset of event). The acceptable duration of prolongation shall be decided upon agreement between the investigator and medical monitor.

b If steroid has been started, its dose shall be tapered to an oral prednisone dose of 10 mg/day or a dose less than that equivalent to this over one month or more before resuming atezolizumab.

c If atezolizumab has been beneficial to subjects, the reintroduction of atezolizumab may be considered after immune-mediated events completely resolved. Atezolizumab treatment may be resumed only after its approval by both investigator (or an appropriate designated person) and medical monitor is documented.

Immune-mediated myocarditis

Immune-mediated myocarditis has occurred in association with atezolizumab treatment. When signs or symptoms (including dyspnea, chest pain, palpitations, fatigue, decreased exercise tolerance and syncope, etc. but not limited to these) suggesting myocarditis are noted, immune-mediated myocarditis should be suspected. Immune-mediated myocarditis shall be differentiated from myocarditis secondary to infection (e.g., mainly viral and in subjects with a recent history of gastrointestinal disease), ischemic events, baseline arrhythmia, the worsening of preexisting heart disease, progression of malignancies, etc.

In all subjects with possible myocarditis, appropriate tests such as cardiac enzyme, electrocardiography, chest radiography, echocardiography and cardiac MRI should be urgently conducted in accordance with guidelines used at the study site. Consult a cardiologist. It should be also considered carrying out an endomyocardial biopsy for a definitive diagnosis and appropriate treatment according to clinical necessity.

Subjects with signs and/or symptoms of myocarditis for whom other causes of disease are not identified should be handled in accordance with guidelines presented in Table 6.

Table 6 Guidelines for the management of immune-mediated myocarditis

Event	Management
Immune-mediated myocarditis, Grade 1	• Refer the subject to a cardiologist.<br>• Start treatment in accordance with guidelines used at the study site.
Immune-mediated myocarditis, Grade 2	• Interrupt atezolizumab treatment for 12 weeks at a maximum after the onset of event[a], and notify it to the medical monitor.<br>• Refer the subject to a cardiologist.<br>• Start treatment in accordance with guidelines used at the study site, and consider antiarrhythmic agents, temporary pacemaker, ECMO or VAD according to needs.<br>• Start treatment with methylprednisolone for intravenous injection at a dose of 1 to 2 mg/kg/day or at an equivalent dose, and switch it to oral prednisone 1 to 2 mg/kg/day or at an equivalent dose if the event improved.<br>• Resume atezolizumab treatment if the event improved to Grade 1 or better[b].<br>• If the event does not improve to Grade 1 or better during atezolizumab treatment interruption, permanently discontinue atezolizumab treatment, and notify it to the medical monitors[c].
immune-mediated myocarditis , Grade 3 to 4	• Permanently discontinue atezolizumab treatment, and notify it to the medical monitors[c].<br>• Refer the subject to a cardiologist.<br>• Start treatment in accordance with guidelines used at the study site, and consider antiarrhythmic agents, temporary pacemaker, ECMO or VAD according to needs.<br>• Start treatment with methylprednisolone for intravenous injection at a dose of 1 to 2 mg/kg/day or at an equivalent dose, and switch it to oral prednisone 1 to 2 mg/kg/day or at an equivalent dose if the event improved.<br>• If the event does not improve within 48 hours after the start of steroid therapy, consider adding an immunosuppressive drug.<br>• If the event improved to Grade 1 or better, taper the steroid dose over one month.

ECMO = extracorporeal membrane oxygenation; VAD = ventricular assist device

[a] When it is necessary to reduce the steroid (if started) dose to an oral prednisone dose of 10 mg/day or dose less than that equivalent to this, the period of atezolizumab interruption may be extended (may be more than 12 weeks after the onset of event). The acceptable duration of prolongation shall be decided upon agreement between the investigator and medical monitor.

[b] If steroid has been started, its dose shall be tapered to an oral prednisone dose of 10 mg/day or a dose less than that equivalent to this over one month or more before resuming atezolizumab.

[c] If atezolizumab has been beneficial to subjects, the reintroduction of atezolizumab may be considered after immune-mediated events completely resolved. Atezolizumab treatment may be resumed only after its approval by both investigator (or an appropriate designated person) and medical monitor is documented.

Seto T, *et al. J Immunother Cancer* 2022; 10:e004025. doi: 10.1136/jitc-2021-004025

Infusion reaction-related events

No premedication is allowed at the initial fusion of atezolizumab. However, premedication with antihistamines or antipyretic-analgesic drugs (e.g., acetaminophen) may be given at Cycle 2 or subsequent cycles to subjects who developed infusion reaction-related events during Cycle 1 of atezolizumab. The use of metamizole (Dipyrone) for the treatment of infusion reaction-related events during atezolizumab treatment is prohibited because it may cause agranulocytosis.

Guidelines for management of drug treatment for infusion reaction-related events during Cycle 1 are presented in Table 7. Infusion reaction-related events during Cycle 2 or subsequent cycles should be managed in accordance with guidelines used at the study site.

Table 7 Guidelines for the management of infusion reaction-related events

Event	Management
IRR, Grade 1	• Reduce the infusion rate by half at the onset of event.<br>• After the event resolved, the investigator should check the infusion with a reduced rate continuously for 30 minutes.<br>• If the infusion has been well tolerated during 30 minutes with a reduced rate after the resolution of the event, the infusion rate may be reincreased to the original rate.
IRR, Grade 2	• Discontinue atezolizumab infusion.<br>• Administer active symptomatic treatment (e.g., oral or intravenous administration of antihistamines, antipyretics, glucocorticoid, epinephrine, bronchodilators, and oxygen).<br>• Resume the infusion at a rate half of that at the onset of event if the symptom recovered to a baseline condition<br>• Orally administer an antihistamine, antipyretic and/or analgesic before infusion in the next and subsequent dosing, and carefully monitor IRRs.
IRR, Grade 3 or 4	• Discontinue atezolizumab infusion.<br>• Administer active symptomatic treatment (e.g., oral or intravenous administration of antihistamines, antipyretics, glucocorticoid, epinephrine, bronchodilators, and oxygen).<br>• Permanently discontinue atezolizumab treatment, and notify it to the medical monitors[a].

IRR = infusion reaction-related events

[a] If atezolizumab has been beneficial to subjects, the reintroduction of atezolizumab may be considered after immune-mediated events completely resolved. Atezolizumab treatment may be resumed only after its approval by both investigator (or an appropriate designated person) and medical monitor is documented.

Pancreatic events

Abdominal pain symptoms with increased amylase and lipase suggesting pancreatitis have occurred in association with atezolizumab treatment. Evaluation of pancreatitis should be included in a differential diagnosis of acute abdominal pain. In an appropriate detailed examination, an assessment of obstruction of the pancreatic duct, and serum amylase and

lipase tests should be included. Guidelines for the management of pancreatic events including pancreatitis are presented in Table 8.

Table 8 Guidelines for the management of pancreatic events including pancreatitis

Event	Management
Amylase and/or lipase increased, Grade 2	• Continue atezolizumab treatment.<br>• Monitor amylase and lipase every week.<br>• For persisting increased laboratory values (e.g., more than 3 weeks), consider treatment with oral prednisone10 mg/day or at an equivalent dose.
Amylase and/or lipase increased, Grade 3 or 4	• Interrupt atezolizumab treatment for 12 weeks at a maximum after the onset of event.[a]<br>• Refer the subject to a gastrointestinal specialist.<br>• Monitor amylase and lipase every other day.<br>• Consider treatment with oral prednisone 1 to 2 mg/kg/day or at an equivalent dose if the event improved.<br>• Resume atezolizumab treatment if the event improved to Grade 1 or better[b].<br>• If the event does not improve to Grade 1 or better during atezolizumab treatment interruption, permanently discontinue atezolizumab treatment, and notify it to the medical monitors[c].<br>• If the event recurs, permanently discontinue atezolizumab treatment, and notify it to the medical monitors.
Immune-mediated pancreatitis, Grade 2 or 3	• Interrupt atezolizumab treatment for 12 weeks at a maximum after the onset of event[a].<br>• Refer the subject to a gastrointestinal specialist.<br>• Start treatment with methylprednisolone for intravenous injection at a dose of 1 to 2 mg/kg/day or at an equivalent dose, and switch it to oral prednisone 1 to 2 mg/kg/day or at an equivalent dose if the event improved.<br>• Resume atezolizumab treatment if the event improved to Grade 1 or better.<br>• If the event does not improve to Grade 1 or better during atezolizumab treatment interruption, permanently discontinue atezolizumab treatment, and notify it to the medical monitors[c].<br>• If the event recurs, permanently discontinue atezolizumab treatment, and notify it to the medical monitors.
Immune-mediated pancreatitis, Grade 4	• Permanently discontinue atezolizumab treatment, and notify it to the medical monitors[c].<br>• Refer the subject to a gastrointestinal specialist.<br>• Start treatment with methylprednisolone for intravenous injection at a dose of 1 to 2 mg/kg/day or at an equivalent dose, and switch it to oral prednisone 1 to 2 mg/kg/day or at an equivalent dose if the event improved.<br>• If the event does not improve within 48 hours after the start of steroid therapy, consider adding an immunosuppressive drug.<br>• If the event improved to Grade 1 or better, taper the steroid dose over one month.

GI = gastrointestinal

[a] When it is necessary to reduce the steroid (if started) dose to an oral prednisone dose of 10 mg/day or dose less than that equivalent to this, the period of atezolizumab interruption may be extended (may be more than 12 weeks after the onset of event). The acceptable duration of prolongation shall be

decided upon agreement between the investigator and medical monitor.

[b] If steroid has been started, its dose shall be tapered to an oral prednisone dose of 10 mg/day or a dose less than that equivalent to this over one month or more before resuming atezolizumab.

[c] If atezolizumab has been beneficial to subjects, the reintroduction of atezolizumab may be considered after immune-mediated events completely resolved. Atezolizumab treatment may be resumed only after its approval by both investigator (or an appropriate designated person) and medical monitor is documented.

Skin events

Rash has occurred in association with atezolizumab treatment. The majority of rash is mild and spontaneously cures and may be with or without pruritus. Persistent and/or severe rash or pruritus should be treated by dermatologists if noted. The implementation of a biopsy should be considered if it is not contraindicated. Guidelines for the management of skin events are presented in Table 9.

Table 9 Guidelines for the management of skin events

Event	Management
Skin events, Grade 1	• Continue atezolizumab treatment.<br>• Consider treatment with local steroid and/or symptomatic treatment (e.g., antihistamine).
Skin events, Grade 2	• Continue atezolizumab treatment.<br>• Consider referring the subject to a dermatologist.<br>• Start treatment with local steroid.<br>• Consider treatment with stronger local steroid if the event does not improve.
Skin events, Grade 3	• Interrupt atezolizumab treatment for 12 weeks at a maximum after the onset of event[a].<br>• Refer the subject to a dermatologist.<br>• Start administration of oral prednisone 10 mg/day or at an equivalent dose. If the event does not improve within 48 to 72 hours, increase the dose to 1 to 2 mg/kg/day.<br>• Resume atezolizumab treatment if the event improved to Grade 1 or better[b].<br>• If the event does not improve to Grade 1 or better during atezolizumab treatment interruption, permanently discontinue atezolizumab treatment, and notify it to the medical monitors[c].
Skin events, Grade 4	• Permanently discontinue atezolizumab treatment, and notify it to the medical monitors.

[a] When it is necessary to reduce the steroid (if started) dose to an oral prednisone dose of 10 mg/day or dose less than that equivalent to this, the period of atezolizumab interruption may be extended (may be more than 12 weeks after the onset of event). The acceptable duration of prolongation shall be decided upon agreement between the investigator and medical monitor.

[b] If steroid has been started, its dose shall be tapered to an oral prednisone dose of 10 mg/day or a dose less than that equivalent to this over one month or more before resuming atezolizumab.

[c] If atezolizumab has been beneficial to subjects, the reintroduction of atezolizumab may be considered after immune-mediated events completely resolved. Atezolizumab treatment may be resumed only after its approval by both investigator (or an appropriate designated person) and medical monitor is documented.

Nervous system disorders

Myasthenia gravis and Guillain-Barre syndrome have occurred when atezolizumab was administered alone. Subjects may develop signs or symptoms of sensory/motor neuron disorders. A detailed diagnostic examination is essential for accurately differentiating from other causes of disease. Guidelines for the management of Nervous system disorders are presented in Table 10.

Table 10 Guidelines for the management of nervous system disorders

Event	Management
Immune-mediated nervous system disorders, Grade 1	• Continue atezolizumab treatment.<br>• Examine the cause of disease.
Immune-mediated nervous system disorders, Grade 2	• Interrupt atezolizumab treatment for 12 weeks at a maximum after the onset of event[a].<br>• Examine the cause of disease.<br>• Start treatment in accordance with guidelines used at the study site.<br>• Resume atezolizumab treatment if the event improved to Grade 1 or better[b].<br>• If the event does not improve to Grade 1 or better during atezolizumab treatment interruption, permanently discontinue atezolizumab treatment, and notify it to the medical monitors[c].
Immune-mediated nervous system disorders, Grade 3 or 4	• Permanently discontinue atezolizumab treatment, and notify it to the medical monitors[c].<br>• Start treatment in accordance with guidelines used at the study site.
Myasthenia gravis and Guillain-Barre syndrome, (any grade)	• Permanently discontinue atezolizumab treatment, and notify it to the medical monitors[c].<br>• Refer the subject to a neurologist.<br>• Start treatment in accordance with guidelines used at the study site.<br>• Consider starting treatment with oral or intravenous prednisone 1 to 2 mg/kg/day or at an equivalent dose.

a When it is necessary to reduce the steroid (if started) dose to an oral prednisone dose of 10 mg/day or dose less than that equivalent to this, the period of atezolizumab interruption may be extended (may be more than 12 weeks after the onset of event). The acceptable duration of prolongation shall be decided upon agreement between the investigator and medical monitor.

b If steroid has been started, its dose shall be tapered to an oral prednisone dose of 10 mg/day or a dose less than that equivalent to this over one month or more before resuming atezolizumab.

c If atezolizumab has been beneficial to subjects, the reintroduction of atezolizumab may be considered after immune-mediated events completely resolved. Atezolizumab treatment may be resumed only after its approval by both investigator (or an appropriate designated person) and medical monitor is documented.

Immune-mediated meningoencephalitis

Immune-mediated meningoencephalitis is an identified risk associated with atezolizumab

treatment. If signs and/or symptoms (including headache, neck pain, confusion, seizure, motor or sensory dysfunction, and changes or decreases in the level of consciousness but not limited to these) suggesting meningitis or encephalitis are noted, immune-mediated meningoencephalitis should be suspected. Encephalopathy due to metabolic imbalance or electrolyte abnormality needs to be differentiated from meningoencephalitis caused by (bacterial, viral or fungal) infection or the progression of malignancies, or meningoencephalitis secondary to tumor-associated processes.

In all subjects with possible meningoencephalitis, brain CT and/or MRI scans should be urgently performed to examine the presence or absence of metastasis, inflammation or edema. If a treating physician judged safe, lumbar puncture should be carried out, and he/she should consult a neurologist.

Subjects with signs and/or symptoms of meningoencephalitis for whom other causes of disease are not identified should be handled in accordance with guidelines presented in Table 11.

Table 11 Guidelines for the management of immune-mediated meningoencephalitis

Event	Management
Immune-mediated meningoencephalitis, any grade	• Permanently discontinue atezolizumab treatment, and notify it to the medical monitors[a].<br>• Refer the subject to a neurologist.<br>• Start treatment with methylprednisolone for intravenous injection at a dose of 1 to 2 mg/kg/day or at an equivalent dose, and switch it to oral prednisone 1 to 2 mg/kg/day or at an equivalent dose if the event improved.<br>• If the event does not improve within 48 hours after the start of steroid therapy, consider adding an immunosuppressive drug.<br>• If the event improved to Grade 1 or better, taper the steroid dose over one month.

[a] If atezolizumab has been beneficial to subjects, the reintroduction of atezolizumab may be considered after immune-mediated events completely resolved. Atezolizumab treatment may be resumed only after its approval by both investigator (or an appropriate designated person) and medical monitor is documented.

Seto T, *et al. J Immunother Cancer* 2022; 10:e004025. doi: 10.1136/jitc-2021-004025
